# Blinded and unblinded sample size reestimation procedures for stepped‐wedge cluster randomized trials

**DOI:** 10.1002/bimj.201700125

**Published:** 2018-08-03

**Authors:** Michael J. Grayling, Adrian P. Mander, James M. S. Wason

**Affiliations:** ^1^ MRC Biostatistics Unit Cambridge Institute of Public Health, Forvie Site, Robinson Way Cambridge Biomedical Campus Cambridge UK; ^2^ Institute of Health and Society Newcastle University Baddiley‐Clark Building Newcastle upon Tyne UK

**Keywords:** blinded, cluster randomized trial, internal pilot, sample size re‐estimation, stepped‐wedge

## Abstract

The ability to accurately estimate the sample size required by a stepped‐wedge (SW) cluster randomized trial (CRT) routinely depends upon the specification of several nuisance parameters. If these parameters are misspecified, the trial could be overpowered, leading to increased cost, or underpowered, enhancing the likelihood of a false negative. We address this issue here for cross‐sectional SW‐CRTs, analyzed with a particular linear‐mixed model, by proposing methods for blinded and unblinded sample size reestimation (SSRE). First, blinded estimators for the variance parameters of a SW‐CRT analyzed using the Hussey and Hughes model are derived. Following this, procedures for blinded and unblinded SSRE after any time period in a SW‐CRT are detailed. The performance of these procedures is then examined and contrasted using two example trial design scenarios. We find that if the two key variance parameters were underspecified by 50%, the SSRE procedures were able to increase power over the conventional SW‐CRT design by up to 41%, resulting in an empirical power above the desired level. Thus, though there are practical issues to consider, the performance of the procedures means researchers should consider incorporating SSRE in to future SW‐CRTs.

## 1 INTRODUCTION

A stepped‐wedge (SW) cluster randomised trial (CRT) involves the sequential roll‐out of an intervention across several clusters over multiple time periods, with the time period in which a cluster begins receiving the intervention determined at random. Recent papers have established methods for sample size determination in the case of cross‐sectional (Hussey & Hughes, 2007) and cohort (Hooper, Teerenstra, de Hoop, & Eldridge, 2016) designs, for trials with multiple levels of clustering and for incomplete block SW‐CRTs (Hemming, Lilford, & Girling, 2015).

Undeniably, there has been a growing interest in the design, and in particular, it has now become associated with scenarios in which there is a belief that the trial's experimental intervention will be effective (Brown & Lilford, 2006; Mdege, Man, Taylor (nee Brown), & Torgerson, 2011). Given this commonly held belief, it may come as a surprise that a recent literature review determined that in 31% of the SW‐CRTs completed by February 2015, there was no significant effect of the experimental intervention on any of the trials primary outcome measures (Grayling, Wason, & Mander, 2017a). To guard against this, implicitly assuming this failure rate was due to the experimental interventions being futile, methodology for the incorporation of interim analyses in SW‐CRTs was recently described (Grayling, Wason, & Mander, 2017b). One other possible explanation is that the studies have been false negatives. A high false‐negative rate could be associated with SW‐CRTs having been underpowered. Methodology available to determine the sample size required by SW‐CRTs is dependent upon the specification of the values of several nuisance parameters (e.g., the between cluster and residual variances). In practice, it may be difficult to provide accurate estimates for these factors, and their misspecification may be leading to under‐powered studies. Alternatively, if these parameters are being misspecified such that SW‐CRTs have been overpowered, there may have been more measurements taken than actually required, leading to unnecessary cost.

A common approach to addressing the specification of nuisance parameters in the trial design literature is the use of a sample size reestimation (SSRE) procedure. Each such method has essentially the same intention: to alleviate the issue of prespecifying nuisance parameters by allowing them to be reestimated during the trial, and the required sample size adjusted. Reviews of SSRE methodology have been provided by Friede and Kieser (2013), Proschan (2009), and Pritchett et al. (2015), among others. We refer the reader to these articles for a wider overview of SSRE. Of importance here is that, broadly speaking, SSRE procedures can be subcategorized according to whether they are a blinded or unblinded technique, and also in relation to whether they reestimate the variance parameters, the effect‐size, or some combination of the two. Regulatory agencies prefer that the blind be maintained whenever possible, so as to not risk compromising the validity of the trial (ICH, 1998). Fortunately therefore, blinded SSRE methodology is today available for a range of settings (e.g. Friede & Kieser, 2013; Golkowski, Friede, & Kieser, 2014; and Kunz, Stallard, Parsons, Todd, & Friede, 2017), with each such procedure typically conferring highly desirable trial operating characteristics.

However, while some results exist on SSRE in multicentre (Jensen & Kieser, 2010) and parallel group CRTs (van Schie & Moerbeek, 2014), no work has established methodology for SSRE in SW‐CRTs, with the increased complexity in the design of SW‐CRTs necessitating a specialised approach. In this article, we address this by developing and exploring the performance of both blinded and unblinded SSRE procedures for cross‐sectional SW‐CRTs. Precisely, we consider the case in which the variance parameters required for sample size determination are to be reestimated, but the effect‐size is prespecified and remains unmodified. Accordingly, we assume that a commonly considered linear‐mixed model will be utilized for data analysis, and develop blinded estimators of the associated key variance parameters. The performance of a SSRE procedure based on these blinded estimators is then compared to an unblinded approach, as a function of their various control parameters, and the parameters of the underlying model. We conclude with a discussion of possible extensions to our approach, and by detailing logistical factors that must be considered when incorporating SSRE in to SW‐CRTs.

## 2 METHODS

### 2.1 Notation, hypotheses, and analysis

We suppose that a cross‐sectional SW‐CRT is to be carried out in *C* clusters over *T* time periods, with *n* individuals recruited per cluster per time period. That is, we assume data will be accrued on new patients in each cluster in each time period. We do not restrict our attention to “balanced” SW‐CRTs however; clusters need not start in the control condition, conclude in the experimental condition, and there does not need to be an equal number of clusters switching to the experimental intervention in each time period.

We assume that the accumulated data will be normally distributed, and the following linear‐mixed model will be utilized for data analysis, as proposed by Hussey and Hughes (2007)

(1) $$Y_{ijk}=\mu +\pi_{j}+\tau X_{ij}+c_{i}+\varepsilon_{ijk}.$$

Here

- $Y_{ijk}$ is the response of the *k*‐th individual (k=1,⋯,n), in the *i*‐th cluster (i=1,⋯,C), in the *j*‐th time period (j=1,⋯,T);
- μ is an intercept term;
- $\pi_{j}$ is a fixed effect for the *j*‐th time period (with $\pi_{1}=0$ for identifiability);
- τ is a fixed treatment effect for the experimental intervention relative to the control;
- $X_{ij}$ is the binary treatment indicator for the *i*‐th cluster and *j*‐th time period. That is, $X_{ij}=1$ if cluster *i* receives the intervention in time period *j*. We denote by *X*, dim(X)=C×T, the matrix formed from the $X_{ij}$. Similarly, we define $X^{\left(t\right)}$, $\text{dim}(X^{\left(t\right)})=C\times t$, to be the matrix formed from the first *t* columns of *X*. That is, $X_{ij}^{\left(t\right)}=X_{ij}$ for i=1,⋯,C and j=1,⋯,t;
- $c_{i}∼N\left(0,\sigma_{c}^{2}\right)$ is a random effect for cluster *i*;
- $\varepsilon_{ijk}∼N\left(0,\sigma_{e}^{2}\right)$ is the individual‐level error.

We denote the vector of fixed effects by $\theta ={\left(\mu ,\pi_{2},\cdots ,\pi_{T},\tau \right)}^{⊤}$. Moreover, we indicate the design matrix linking ***θ*** to the vector of responses $Y_{T,n}$, from the set of time periods T, given an allocation matrix *X*, and a per cluster per period sample size of *n*, by $D_{T,n}$. We similarly denote the covariance matrix of $Y_{T,n}$, given $\sigma_{c}^{2}$ and $\sigma_{e}^{2}$, by $\text{Cov}\left(Y_{T,n},Y_{T,n}|\sigma_{c}^{2},\sigma_{e}^{2}\right)=\Sigma_{T,n,\sigma_{c}^{2},\sigma_{e}^{2}}$. As is noted in Hussey and Hughes (2007), by the above choice of linear‐mixed model, $\Sigma_{T,n,\sigma_{c}^{2},\sigma_{e}^{2}}$ is a C|T|n×C|T|n block diagonal matrix. Each block, corresponding to the responses from each cluster, is of dimension |T|n×|T|n. Precisely, it is given by $\sigma_{e}^{2}I_{|T|n}+\sigma_{c}^{2}J_{|T|n}$, where $I_{m}$ and $J_{m}$ are the m×m identity and unit matrices, respectively.

We perform a one‐sided hypothesis test for τ

$$H_{0}:\tau \leq 0,\;H_{1}:\tau >0,$$

and assume that it is desired to have a type‐I error rate of α when τ=0, and to have power to reject *H*_0_ of 1−β when τ=δ, for some specified δ>0. Note that SSRE procedures for two‐sided hypotheses are also easily achievable.

Finally, we assume that hypothesized values for the variance parameters $\sigma_{c}^{2}$ and $\sigma_{e}^{2}$ have been provided, which we denote by ${\tilde{\sigma }}_{c}^{2}$ and ${\tilde{\sigma }}_{e}^{2}$. Alternatively, a value for one of these parameters, and a value for the intracluster correlation (ICC) ρ, $\tilde{\rho }={\tilde{\sigma }}_{c}^{2}/\left({\tilde{\sigma }}_{c}^{2}+{\tilde{\sigma }}_{e}^{2}\right)$, could be specified, such that ${\tilde{\sigma }}_{c}^{2}$ and ${\tilde{\sigma }}_{e}^{2}$ can still be determined. Given these values, we assume a sample size calculation has been performed (using the methods to be described shortly) and values for *X* and *n* (and thus also *C* and *T* since dim(X)=C×T) have subsequently been specified. For reasons to be elucidated below, we refer to this *n* as $n_{\text{init}}$.

With the above, a conventional SW‐CRT can be conducted as follows. We recruit $n_{\text{init}}$ individuals per cluster per time period, with the experimental intervention allocated according to the matrix *X*. On completion, we use restricted error maximum likelihood (REML) estimation to acquire an estimate of $\hat{\tau }$, denoted $\hat{\tau }$, and a value for $\hat{I}={\left\{\text{Var}\left(\hat{\tau }\right)\right\}}^{-1}$. Next, we compute the test statistic $T=\hat{\tau }{\hat{I}}^{1/2}$, and reject *H*_0_ if T>e, where *e* is the solution to

$$\begin{array}{cc} \alpha & =\int_{e}^{\infty }\varphi \left\{x,0,1,\nu \right\}dx,\\ \nu & =n_{\text{init}}CT-C-T. \end{array}$$

Here, φ{x,μ,Λ,ν} is the probability density function of a *t*‐distribution with mean μ, covariance Λ, and degrees of freedom ν, evaluated at *x*. Specifically, we take ν to be the degrees of freedom in a corresponding balanced multilevel ANOVA design. Later, we will discuss the implications of this, and describe other possible ways to prescribe ν.

We next detail how the above can be extended to allow SSRE to be incorporated in to the design.

### 2.2 Sample size reestimation procedures

A single interim analysis for SSRE is included in the SW‐CRT design after a designated time period t∈{1,⋯,T−1}. Precisely, we assume that the trial is conducted as per matrix *X* and the value $n_{\text{init}}$ for time periods 1,⋯,t. After this, we compute estimates for the variance parameters, ${\hat{\sigma }}_{c}^{2}$ and ${\hat{\sigma }}_{e}^{2}$, based upon the accumulated data. Below, we detail how exactly this is achieved in the blinded and unblinded procedures. Here, we discuss how these estimates are then used. Note that, as was discussed in Section 1, we are reestimating only $\sigma_{c}^{2}$ and $\sigma_{e}^{2}$: the effect size δ will not be reestimated.

We search numerically as follows to determine the required per cluster per period sample size for the remainder of the trial, $n_{\text{reest}}$, to convey the desired power if $\sigma_{c}^{2}={\hat{\sigma }}_{c}^{2}$ and $\sigma_{e}^{2}={\hat{\sigma }}_{e}^{2}$. Thus, the number of clusters remains fixed throughout the trial; it is the per cluster per period sample size that is adjusted. We consider possible alternatives to this in the discussion.

Firstly, suppose $n_{\text{reest}}$ has been chosen, then time periods t+1,⋯,T of the trial are conducted using the matrix *X* for treatment allocation, and recruiting $n_{\text{reest}}$ individuals per cluster per period. At the end of the trial the linear‐mixed model (1) with REML estimation are utilized to acquire $\hat{\tau }$ and $\hat{I}$ as above. The test statistic $T=\hat{\tau }{\hat{I}}^{1/2}$ is again determined, and *H*_0_ rejected if T>e, but *e* is now the solution to

$$\begin{array}{cc} \alpha & =\int_{e}^{\infty }\varphi \left\{x,0,1,\nu_{n_{\text{reest}}}\right\}dx,\\ \nu_{n_{\text{reest}}} & =n_{\text{init}}Ct+n_{\text{reest}}C\left(T-t\right)-C-T. \end{array}$$

Here, $\nu_{n_{\text{reest}}}$ is the degrees of freedom in a balanced multilevel ANOVA design if a sample size of $n_{\text{init}}$ is used per cluster per period in time periods 1,⋯,t, and a sample size of $n_{\text{reest}}$ is used per cluster per period in time periods t+1,⋯,T.

The power to reject *H*_0_ when τ=δ, for a particular $n_{\text{reest}}$, can thus be estimated at the interim as

$$\text{P}\left(\text{Reject}\;H_{0}|n_{\text{reest}}\right)=\int_{e}^{\infty }\varphi \left\{x,\delta I^{1/2},1,\nu_{n_{\text{reest}}}\right\}dx,$$

where *I* is given by the inverse of element [T+1,T+1] of the following matrix

$$\begin{array}{cc} & \left(D_{\left\{1,\cdots ,t\right\},n_{\text{init}}}^{T}\Sigma_{\left\{1,\cdots ,t\right\},n_{\text{init}},{\hat{\sigma }}_{c}^{2},{\hat{\sigma }}_{e}^{2}}^{-1}D_{\left\{1,\cdots ,t\right\},n_{\text{init}}}+\right.\\ & \;\;{\left.+D_{\left\{t+1,\cdots ,T\right\},n_{\text{reest}}}^{T}\Sigma_{\left\{t+1,\cdots ,T\right\},n_{\text{reest}},{\hat{\sigma }}_{c}^{2},{\hat{\sigma }}_{e}^{2}}^{-1}D_{\left\{t+1,\cdots ,T\right\},n_{\text{reest}}}\right)}^{-1}. \end{array}$$

This matrix arises as the theoretical covariance matrix of the maximum likelihood estimator of ***θ*** when a sample size of $n_{\text{init}}$ is used per cluster per period in time periods 1,⋯,t, and a sample size of $n_{\text{reest}}$ is used per cluster per period in time periods t+1,⋯,T (see, e.g. Fitzmaurice, Laird, & Ware (2011) for details on how this form of covariance matrix is constructed).

Therefore, we can compute the required value for $n_{\text{reest}}$ by searching for the minimal integer solution to the following equation

$$\text{P}(\text{Reject}\;H_{0}|n_{\text{reest}})\geq 1-\beta .$$

In fact, to make our SSRE procedures more applicable in practice, and to guard against unrealistically large values for $n_{\text{reest}}$, we carry out the remaining periods of the trial recruiting $n_{\text{final}}$ individuals per cluster per time period, where

$$n_{\text{final}}=\left\{\begin{array}{cc} n_{\text{min}} & :n_{\text{reest}}<n_{\text{min}},\\ n_{\text{reest}} & :n_{\text{min}}\leq n_{\text{reest}}\leq n_{\text{max}},\\ n_{\text{max}} & :n_{\text{max}}<n_{\text{reest}}. \end{array}\right.$$

Here, $n_{\text{min}}\in N^{+}$ and $n_{\text{max}}\in N^{+}$, with $n_{\text{min}}<n_{\text{max}}$, are designated values for the minimal and maximal allowed number of patients per cluster per period following the reestimation. There are various possible approaches to how values for $n_{\text{min}}$ and $n_{\text{max}}$ can be specified. Wittes and Brittain (1990) advocated for the initially planned sample size to never be reduced. Here, this would correspond to $n_{\text{min}}=n_{\text{init}}$. In contrast, Birkett and Day (1994) proposed that the initially planned sample size should be reduced. Gould (1992) recommended that a realistic maximum sample size should be specified. In practice, the values of $n_{\text{min}}$ and $n_{\text{max}}$ may need to be chosen based upon the potentially attainable values of *n* in a particular trial scenario.

Finally, following determination of $n_{\text{final}}$, the remainder of the trial and ensuant analysis is conducted as described above, to determine whether to reject *H*_0_.

Note that the sample size required by a classical fixed sample SW‐CRT design, given an allocation matrix *X*, can be determined using the above by treating $n_{\text{init}}$ as a variable rather than a fixed parameter, and searching for the minimal $n_{\text{init}}$ such that $\text{P}(\text{Reject}\;H_{0}|0)\geq 1-\beta$ when t=T. Alternatively, $n_{\text{init}}$ could be specified and the matrix *X* determined for the desired power.

All that remains to be elucidated in the above procedure is the means of determining the estimates ${\hat{\sigma }}_{c}^{2}$ and ${\hat{\sigma }}_{e}^{2}$. As discussed, we describe both blinded and unblinded approaches to their specification.

The unblinded procedure is as follows. After time period *t*, we fit the following model to the accumulated data using REML estimation

$$Y_{ijk}=\left\{\begin{array}{cc} \mu +c_{i}+\pi_{j}+X_{ij}\tau +\varepsilon_{ijk} & :\text{if}\;1_{t}^{⊤}X^{\left(t\right)}1_{t}>0\;\text{and}\;t>1,\\ \mu +c_{i}+\pi_{j}+\varepsilon_{ijk} & :\text{if}\;1_{t}^{⊤}X^{\left(t\right)}1_{t}=0\;\text{and}\;t>1,\\ \mu +c_{i}+X_{ij}\tau +\varepsilon_{ijk} & :\text{if}\;1_{t}^{⊤}X^{\left(t\right)}1_{t}>0\;\text{and}\;t=1,\\ \mu +c_{i}+\varepsilon_{ijk} & :\text{if}\;1_{t}^{⊤}X^{\left(t\right)}1_{t}=0\;\text{and}\;t=1. \end{array}\right..$$

Here, $1_{t}={\left(1,\cdots ,1\right)}^{⊤}$, with $\dim(1_{t})=t\times 1$. In the above, $1_{t}^{⊤}X^{\left(t\right)}1_{t}>0$ is included as a qualifier to indicate the term $X_{ij}\tau$ should appear in our model as at least one cluster has been administered the experimental intervention in some time period. Similarly, t>1 indicates period effects should be accounted for in the model. Following REML estimation, we attain our values for ${\hat{\sigma }}_{c}^{2}$ and ${\hat{\sigma }}_{e}^{2}$ immediately, and use them in the above algorithm to determine $n_{\text{final}}$.

For the blinded procedure, it is first useful to note precisely what we mean by “blinded.” Here, it refers to the fact that the treatment indicator of an observation is not known, but that it is known which observations belong to the sample cluster and time period. With this, define

$$\begin{array}{cc} {\bar{S}}_{Ct-t}^{2} & =\frac{n}{Ct-t}\sum_{i=1}^{C}\sum_{j=1}^{t}{\left({\bar{Y}}_{ij.}-{\bar{Y}}_{.j.}\right)}^{2},\\ S_{Ct}^{2} & =\frac{1}{nCt-Ct}\sum_{i=1}^{C}\sum_{j=1}^{t}\sum_{k=1}^{n_{\text{init}}}{\left(Y_{ijk}-{\bar{Y}}_{ij.}\right)}^{2}, \end{array}$$

where

$$\begin{array}{cc} {\bar{Y}}_{.j.} & =\frac{1}{n_{\text{init}}C}\sum_{i=1}^{C}\sum_{k=1}^{n_{\text{init}}}Y_{ijk},\\ {\bar{Y}}_{ij.} & =\frac{1}{n_{\text{init}}}\sum_{k=1}^{n_{\text{init}}}Y_{ijk}. \end{array}$$

In the Supplementary Material we derive that

$$\begin{array}{cc} \text{E}({\bar{S}}_{Ct-t}^{2}) & =\sigma_{e}^{2}+n_{\text{init}}\sigma_{c}^{2}+\frac{n_{\text{init}}\tau^{2}}{Ct-t}1_{t}^{⊤}X^{\left(t\right)}1_{t}-\frac{n_{\text{init}}\tau^{2}}{C(Ct-t)}\left(1_{t}^{⊤}X^{\left(t\right)}\right)\cdot \left(1_{t}^{⊤}X^{\left(t\right)}\right),\\ \text{E}(S_{Ct}^{2}) & =\sigma_{e}^{2}. \end{array}$$

Given a particular choice for τ in the above, which we shall denote $\tau_{*}$, these equations are used to estimate $\sigma_{c}^{2}$ and $\sigma_{e}^{2}$ as follows

- Compute ${\bar{S}}_{Ct-t}^{2}$ and $S_{Ct}^{2}$ using the formulae above and the accrued data.
- Define $f({\bar{S}}_{Ct-t}^{2},\sigma_{e}^{2},X^{\left(t\right)},n,\tau )$ as
  $$\begin{array}{cc} f({\bar{S}}_{Ct-t}^{2},\sigma_{e}^{2},X^{\left(t\right)},n,\tau ) & =\frac{1}{n}\left\{{\bar{S}}_{Ct-t}^{2}-\sigma_{e}^{2}-\frac{n\tau^{2}}{Ct-t}1_{t}^{⊤}X^{\left(t\right)}1_{t}\;+\right.\\ & \;\;\left.+\;\frac{n\tau^{2}}{C(Ct-t)}\left(1_{t}^{⊤}X^{\left(t\right)}\right)\cdot \left(1_{t}^{⊤}X^{\left(t\right)}\right)\right\}. \end{array}$$
- Set ${\hat{\sigma }}_{e}^{2}=S_{Ct}^{2}$ and
  $${\hat{\sigma }}_{c}^{2}=\left\{\begin{array}{cc} f({\bar{S}}_{Ct-t}^{2},{\hat{\sigma }}_{e}^{2},X^{\left(t\right)},n_{\text{init}},\tau_{*}) & :\text{if}\;f({\bar{S}}_{Ct-t}^{2},{\hat{\sigma }}_{e}^{2},X^{\left(t\right)},n_{\text{init}},\tau_{*})>0,\\ f({\bar{S}}_{Ct-t}^{2},{\hat{\sigma }}_{e}^{2},X^{\left(t\right)},n_{\text{init}},0) & :\text{if}\;f\left({\bar{S}}_{Ct-t}^{2},{\hat{\sigma }}_{e}^{2},X^{\left(t\right)},n_{\text{init}},\tau_{*}\right)<0<f\left({\bar{S}}_{Ct-t}^{2},{\hat{\sigma }}_{e}^{2},X^{\left(t\right)},n_{\text{init}},0\right),\\ 0 & :\text{otherwise}. \end{array}\right.$$
  Here, our specification of ${\hat{\sigma }}_{c}^{2}$ allows us to make use of $\tau_{*}$ when it will give rise to a value of ${\hat{\sigma }}_{c}^{2}$ such that ${\hat{\sigma }}_{c}^{2}>0$, but assists against overcorrection for a nonzero treatment effect by specifying ${\hat{\sigma }}_{c}^{2}=f\left({\bar{S}}_{Ct-t}^{2},{\hat{\sigma }}_{e}^{2},X^{\left(t\right)},n_{\text{init}},0\right)$ when $f\left({\bar{S}}_{Ct-t}^{2},{\hat{\sigma }}_{e}^{2},X^{\left(t\right)},n_{\text{init}},\tau_{*}\right)<0<f\left({\bar{S}}_{Ct-t}^{2},{\hat{\sigma }}_{e}^{2},X^{\left(t\right)},n_{\text{init}},0\right)$.

We then utilize the algorithm from earlier to determine the value of $n_{\text{final}}$.

Note that if $\tau_{*}=\tau$, the above are unbiased estimators for the variance parameters $\sigma_{c}^{2}$ and $\sigma_{e}^{2}$.

For further clarity, the full unblinded and blinded SSRE procedures are detailed algorithmically in the Supplementary Material. In addition, we also discuss in the Supplementary Material possible alternative methods for reestimating the between cluster and residual variances in a blinded manner, and why we believe our chosen approach should be preferred.

### 2.3 Simulation study

With the above considerations, a SSRE trial design scenario is fully specified given D, where

$$D=\left\{X,t,\sigma_{c}^{2},\sigma_{e}^{2},{\tilde{\sigma }}_{c}^{2},{\tilde{\sigma }}_{e}^{2},\alpha ,\beta ,\delta ,\mu ,\pi ,\tau ,n_{\text{min}},n_{\text{max}},B\right\}\cup I_{\left\{B=1\right\}}\left\{\tau_{*}\right\}.$$

Here, $\pi ={\left(\pi_{2},\cdots ,\pi_{T}\right)}^{T}$ is the vector of period effects, and *B* is a binary indicator variable that takes the value 1 if blinded SSRE is utilized, and the value 0 if unblinded SSRE is utilized. Finally, $I_{A}$ is the indicator function on event A.

Given D we can simulate a SW‐CRT utilizing this SSRE procedure by generating random multivariate normal observations. From this, the empirical rejection rate (ERR) of a particular scenario can be estimated by performing a large number of replicates simulations. To this end, define $R_{s}\left(D\right)$ to be 1 if the result of replicate *s* of a trial simulated according to scenario D is to reject *H*_0_, and 0 otherwise. For any number of replicates *r*, the ERR for scenario D is

$$ERR\left(D\right)=\frac{1}{r}\sum_{s=1}^{r}R_{s}\left(D\right).$$

Similarly, we record the values of ${\hat{N}}_{s}\left(D\right)=n_{\text{s,init}}Ct+n_{\text{s,final}}C\left(T-t\right)$, the total sample size required in replicate *s*, computed using $n_{\text{s,init}}$ and $n_{\text{s,final}}$, the initial and reestimated per cluster per period sample sizes for this replicate. This allows us to examine the distribution of the total required sample size, which we denote from here by $\hat{N}=\hat{N}\left(D\right)$. In this article, $r=10^{5}$ for all considered scenarios.

In what follows, we consider performance in a wide variety of scenarios. However, many of the parameters in D remain fixed. In particular, they are set based on two motivating trial design scenarios.

Firstly, Bashour et al. (2013) conducted a SW‐CRT to assess the effect of training doctors in communication skills on women's satisfaction with doctor‐woman relationship during labour and delivery. The trial utilized a balanced complete block SW‐CRT design, enrolling four hospitals, and gathering data over five time periods. The final analysis estimated the between cluster and residual variances to be $\sigma_{c}^{2}=0.02$ and $\sigma_{e}^{2}=0.51$, respectively. For these variance parameters, the utilised design would have required 70 patients per cluster per time period for the trials desired type‐I and type‐II error rates of 0.05 and 0.1, respectively, when powering for a clinically relevant difference of 0.2, using the methods above. Thus in Trial Design Setting (TDS) 1 we fix $\sigma_{c}^{2}=0.02$, $\sigma_{e}^{2}=0.51$, α=0.05, β=0.1, and δ=0.2. Moreover, C=4, T=5, and *X* is such that a single cluster switches to the experimental intervention in time periods two through five.

The parameters of TDS2 are based upon the typical characteristics of SW‐CRTs according to a recent review (Grayling et al., 2017a). Precisely, adapting Grayling et al. (2017b), we set $\sigma_{c}^{2}=1/9$ and $\sigma_{e}^{2}=1$, in order to consider a more modest value for the ICC of ρ=0.1. In addition, we set α=0.025, β=0.2, δ=0.267, C=20, T=9, and *X* such that three clusters switch to the experimental intervention in time periods two through five, and two clusters in time periods six through nine. This implies that the actual total sample size required by this trial is approximately that accrued on average in SW‐CRTs completed to date.

For simplicity, in both TDSs we take $\mu =\pi_{2}=\cdots =\pi_{T}=0$. We therefore consider the effect of different choices for *t*, ${\tilde{\sigma }}_{c}^{2}$, ${\tilde{\sigma }}_{e}^{2}$, $n_{\text{min}}$, $n_{\text{max}}$, τ, $\tau_{*}$, and *B*. We in general assume that $\left({\tilde{\sigma }}_{c}^{2},{\tilde{\sigma }}_{e}^{2}\right)\in \left\{0.5\sigma_{c}^{2},\sigma_{c}^{2},1.5\sigma_{c}^{2}\right\}\times \left\{0.5\sigma_{e}^{2},\sigma_{e}^{2},1.5\sigma_{e}^{2}\right\}$. That is, each variance component is either underspecified by 50%, correctly specified, or overspecified by 50%. Moreover, we consider three possible combinations of values for $n_{\text{min}}$, $n_{\text{max}}$

- When $n_{\text{min}}=1$ and $n_{\text{max}}=$ 1,000, so there is no practical limit on $n_{\text{final}}$.
- When $n_{\text{min}}=n_{\text{init}}$ and $n_{\text{max}}=$ 1,000, so there is no practical upper limit on $n_{\text{final}}$, but it must be at least as large as the initially specified per cluster per period sample size.
- When $n_{\text{min}}=1$ and $n_{\text{max}}=n_{\text{init}}$, so there is no lower limit on $n_{\text{final}}$, but it cannot be larger than the initially specified per cluster per period sample size.

The upper limits of 1,000 are retained simply for computational reasons, as memory allocation issues can occur when $n_{\text{final}}$ is extremely large. Note that given $n_{\text{min}}\geq 1$ in all instances, the trial will never be terminated at the reestimation point, with all clusters recruiting at least one participant in the subsequent time periods. Moreover, results are presented here only for the first scenario with $n_{\text{min}}=1$ and $n_{\text{max}}=$ 1,000. Our findings for the other two scenarios are provided in the Supplementary Material.

Software to perform our simulations is available from https://github.com/mjg211/article_code.

## 3 RESULTS

### 3.1 Performance for varying ${\tilde{\sigma }}_{c}^{2}$ and ${\tilde{\sigma }}_{e}^{2}$

To begin, we consider how the SSRE procedures perform as $\sigma_{c}^{2}$ and $\sigma_{e}^{2}$ are misspecified to varying degrees. Precisely, we set t=3 and t=5 for TDS1 and TDS2, respectively, and explore $\left({\tilde{\sigma }}_{c}^{2},{\tilde{\sigma }}_{e}^{2}\right)\in \left\{0.5\sigma_{c}^{2},\sigma_{c}^{2},1.5\sigma_{c}^{2}\right\}\times \left\{0.5\sigma_{e}^{2},\sigma_{e}^{2},1.5\sigma_{e}^{2}\right\}$, with τ=0; giving the empirical type‐I error rate (ETI), or τ=δ; giving the empirical power (EP). For the blinded procedure, we take $\tau_{*}=0$, and here we consider only the case where $n_{\text{min}}=1$ and $n_{\text{max}}=$ 1,000. Our findings are presented in Table 1, which displays the ERRs of the reestimation procedures and the corresponding fixed sample SW‐CRT design. Furthermore, in Table 2 the median value of $\hat{N}$ in each case is listed. Additionally, Supporting Information Figures 1, 2, 4, and 5 together depict the distributions of ${\hat{\sigma }}_{e}^{2}$ and ${\hat{\sigma }}_{c}^{2}$ when employing blinded or unblinded reestimation, when τ=0 and τ=δ. Similarly, Supporting Information Figures 3 and 6 display the corresponding distributions of $\hat{N}$.

**Table 1:** Empirical type‐I error rates (τ=0) and power (τ=δ) of the blinded ($\tau_{*}=0$) and unblinded reestimation procedures, along with the corresponding fixed sample SW‐CRT design are shown

		τ=0				τ=δ		
${\tilde{\sigma }}_{c}^{2}$	${\tilde{\sigma }}_{e}^{2}$	Blinded	Unblinded	Fixed		Blinded	Unblinded	Fixed
Trial Design Setting 1								
$0.5\sigma_{c}^{2}$	$0.5\sigma_{e}^{2}$	0.0581	0.0576	0.0646		0.8812	0.8799	0.6922
$0.5\sigma_{c}^{2}$	$\sigma_{e}^{2}$	0.0589	0.0601	0.0585		0.8859	0.8858	0.8929
$0.5\sigma_{c}^{2}$	$1.5\sigma_{e}^{2}$	0.0632	0.0641	0.0569		0.8935	0.8963	0.9700
$\sigma_{c}^{2}$	$0.5\sigma_{e}^{2}$	0.0568	0.0563	0.0627		0.8817	0.8788	0.7051
$\sigma_{c}^{2}$	$\sigma_{e}^{2}$	0.0593	0.0619	0.0600		0.8848	0.8843	0.9034
$\sigma_{c}^{2}$	$1.5\sigma_{e}^{2}$	0.0643	0.0630	0.0567		0.8957	0.8968	0.9736
$1.5\sigma_{c}^{2}$	$0.5\sigma_{e}^{2}$	0.0583	0.0564	0.0621		0.8825	0.8804	0.7084
$1.5\sigma_{c}^{2}$	$\sigma_{e}^{2}$	0.0594	0.0587	0.0594		0.8862	0.8864	0.9067
$1.5\sigma_{c}^{2}$	$1.5\sigma_{e}^{2}$	0.0630	0.0642	0.0567		0.8974	0.8954	0.9736
Trial Design Setting 2								
$0.5\sigma_{c}^{2}$	$0.5\sigma_{e}^{2}$	0.0254	0.0270	0.0266		0.8282	0.8002	0.5875
$0.5\sigma_{c}^{2}$	$\sigma_{e}^{2}$	0.0260	0.0261	0.0255		0.8284	0.8068	0.8024
$0.5\sigma_{c}^{2}$	$1.5\sigma_{e}^{2}$	0.0269	0.0261	0.0259		0.8284	0.8094	0.9125
$\sigma_{c}^{2}$	$0.5\sigma_{e}^{2}$	0.0260	0.0263	0.0271		0.8295	0.8006	0.5910
$\sigma_{c}^{2}$	$\sigma_{e}^{2}$	0.0271	0.0274	0.0257		0.8283	0.8059	0.8021
$\sigma_{c}^{2}$	$1.5\sigma_{e}^{2}$	0.0262	0.0274	0.0253		0.8301	0.8123	0.9333
$1.5\sigma_{c}^{2}$	$0.5\sigma_{e}^{2}$	0.0266	0.0273	0.0258		0.8261	0.8026	0.5903
$1.5\sigma_{c}^{2}$	$\sigma_{e}^{2}$	0.0258	0.0258	0.0255		0.8288	0.8053	0.8486
$1.5\sigma_{c}^{2}$	$1.5\sigma_{e}^{2}$	0.0269	0.0268	0.0254		0.8287	0.8095	0.9348

Results are given for Trial Design Settings 1 (t=3) and 2 (t=5), for a selection of possible values for the assumed variance parameters, with $n_{\text{min}}=1$ and $n_{\text{max}}=$ 1,000.

**Table 2:** Median values of the final total required sample size ($\hat{N}$) are shown for τ=0 and τ=δ using the blinded ($\tau_{*}=0$) and unblinded reestimation procedures, along with the corresponding fixed sample SW‐CRT design

		τ=0				τ=δ		
${\tilde{\sigma }}_{c}^{2}$	${\tilde{\sigma }}_{e}^{2}$	Blinded	Unblinded	Fixed		Blinded	Unblinded	Fixed
Trial Design Setting 1								
$0.5\sigma_{c}^{2}$	$0.5\sigma_{e}^{2}$	1,556	1,556	700		1,612	1,556	700
$0.5\sigma_{c}^{2}$	$\sigma_{e}^{2}$	1,364	1,356	1,340		1,396	1,356	1,340
$0.5\sigma_{c}^{2}$	$1.5\sigma_{e}^{2}$	1,376	1,376	2,040		1,408	1,376	2,040
$\sigma_{c}^{2}$	$0.5\sigma_{e}^{2}$	1,552	1,544	720		1,600	1,544	720
$\sigma_{c}^{2}$	$\sigma_{e}^{2}$	1,352	1,352	1,400		1,392	1,352	1,400
$\sigma_{c}^{2}$	$1.5\sigma_{e}^{2}$	1,384	1,384	2,120		1,416	1,384	2,120
$1.5\sigma_{c}^{2}$	$0.5\sigma_{e}^{2}$	1,540	1,532	740		1,596	1,532	740
$1.5\sigma_{c}^{2}$	$\sigma_{e}^{2}$	1,348	1,348	1,420		1,388	1,348	1,420
$1.5\sigma_{c}^{2}$	$1.5\sigma_{e}^{2}$	1,388	1,388	2,140		1,420	1,388	2,140
Trial Design Setting 2								
$0.5\sigma_{c}^{2}$	$0.5\sigma_{e}^{2}$	1,440	1,440	720		1,600	1,440	720
$0.5\sigma_{c}^{2}$	$\sigma_{e}^{2}$	1,260	1,260	1,260		1,340	1,260	1,260
$0.5\sigma_{c}^{2}$	$1.5\sigma_{e}^{2}$	1,240	1,240	1,800		1,320	1,240	1,800
$\sigma_{c}^{2}$	$0.5\sigma_{e}^{2}$	1,440	1,440	720		1,600	1,440	720
$\sigma_{c}^{2}$	$\sigma_{e}^{2}$	1,260	1,260	1,260		1,340	1,260	1,260
$\sigma_{c}^{2}$	$1.5\sigma_{e}^{2}$	1,260	1,260	1,980		1,340	1,260	1,980
$1.5\sigma_{c}^{2}$	$0.5\sigma_{e}^{2}$	1,440	1,440	720		1,600	1,440	720
$1.5\sigma_{c}^{2}$	$\sigma_{e}^{2}$	1,280	1,280	1,440		1,360	1,280	1,440
$1.5\sigma_{c}^{2}$	$1.5\sigma_{e}^{2}$	1,260	1,260	1,980		1,340	1,260	1,980

Results are given for Trial Design Settings 1 (t=3) and 2 (t=5), for a selection of possible values for the assumed variance parameters, with $n_{\text{min}}=1$ and $n_{\text{max}}=$ 1,000.

In general, for the fixed design, assuming larger values for the variance parameters leads to an increased EP, a decreased ETI, and larger requisite final sample sizes, as would be expected. While this appears to be true for the EP in the SSRE designs, it is not always the case for the ETI. However, assuming larger values for the variance parameters does lead to improved performance of the SSRE procedures in terms of estimating $\sigma_{c}^{2}$ and $\sigma_{e}^{2}$ at the interim analysis. This is true both in terms of the median reestimated values, and the observed variability in the estimates (Supporting Information Figures 1, 2, 4, and 5).

It should be noted that, as we would expect, the blinded reestimation procedure tends to perform worse at estimating $\sigma_{c}^{2}$ when τ=δ than τ=0 (Supporting Information Figures 1, 2, 4, and 5), as the blinded estimator is only unbiased when $\tau_{*}=\tau$. Moreover, for τ=δ the unblinded procedure tends to underestimate the value of $\sigma_{c}^{2}$, while the blinded procedure overestimates its value.

In TDS1, for certain values of the assumed variance parameters there is large inflation of the ETI above the nominal level. This is an issue common to both the SSRE procedures and the fixed design, with the maximal inflation observed for ${\hat{\sigma }}_{c}^{2}=0.5\sigma_{c}^{2}$, ${\hat{\sigma }}_{e}^{2}=\sigma_{e}^{2}$ in the unblinded reestimation design, where the ETI is 0.0653. Finally, for TDS1, the blinded and unblinded procedures have similar values for the EP. However, from Table 2 we can say that the blinded procedure routinely requires a larger number of measurements than its unblinded analog.

In contrast, for TDS2 there is only small inflation to the ETI, with the blinded and unblinded methods displaying similar ETIs. In this case, the blinded procedure always has a larger EP than the unblinded procedure. Examining Table 2, it is clear why this is the case, as the blinded procedure has a higher median value for $\hat{N}$, which is in turn a consequence of our observation above on the blinded procedures propensity to overestimate $\sigma_{c}^{2}$. Given that the unblinded procedure always attains the desired power, this is arguably a disadvantage of the blinded approach.

Overall, it is clear that when the value of $\sigma_{e}^{2}$ is underspecified, the SSRE procedures generally have a far higher EP than the corresponding fixed SW‐CRT design, with comparable if not preferable ETIs. For example, when ${\tilde{\sigma }}_{c}^{2}=0.5\sigma_{c}^{2}$ and ${\tilde{\sigma }}_{e}^{2}=0.5\sigma_{e}^{2}$ in TDS2, the blinded procedure has an EP of 0.8282, while the corresponding conventional SW‐CRT design has an EP of only 0.5875; an increase of 41%. The reason for this is clear from Table 2, as we can see the blinded procedure is able to effectively utilize the interim estimates of the variance parameters to increase the final requisite sample size.

Similarly, when the variance parameters are overspecified, the SSRE procedures are able to reduce the total requisite sample size by taking $n_{\text{final}}<n_{\text{init}}$, and bring the EP closer to the desired level.

### 3.2 Performance for varying *t*

Next, we assess the impact upon the ETI and EP of the choice of the SSRE point *t*. As above, we set $\tau_{*}=0$ for the blinded procedure, and take $n_{\text{min}}=1$ and $n_{\text{max}}=$ 1,000. However, we now focus only on the cases where $\left({\tilde{\sigma }}_{c}^{2},{\tilde{\sigma }}_{e}^{2}\right)\in \left\{0.5\left(\sigma_{c}^{2},\sigma_{e}^{2}\right),\left(\sigma_{c}^{2},\sigma_{e}^{2}\right),1.5\left(\sigma_{c}^{2},\sigma_{e}^{2}\right)\right\}$.

Table 3 displays the ETI and EP of the blinded and unblinded SSRE procedures when τ=0 and τ=δ, respectively, for t∈{2,3,4} in TDS1 and t∈{3,5,7} in TDS2. In addition, Table 4 displays the corresponding median values of $\hat{N}$. Finally, Supporting Information Figures 7– 12 display the distributions of ${\hat{\sigma }}_{c}^{2}$, ${\hat{\sigma }}_{e}^{2}$, and $\hat{N}$, for these scenarios.

**Table 3:** Empirical type‐I error rates (τ=0) and power (τ=δ) of the blinded ($\tau_{*}=0$) and unblinded reestimation procedures are shown

			τ=0				τ=δ		
Trial Design Setting 1									
Procedure	${\tilde{\sigma }}_{c}^{2}$	${\tilde{\sigma }}_{e}^{2}$	t=2	t=3	t=4		t=2	t=3	t=4
Blinded	$0.5\sigma_{c}^{2}$	$0.5\sigma_{e}^{2}$	0.0592	0.0581	0.0512		0.8702	0.8812	0.8151
Blinded	$\sigma_{c}^{2}$	$\sigma_{e}^{2}$	0.0597	0.0593	0.0617		0.8851	0.8848	0.8971
Blinded	$1.5\sigma_{c}^{2}$	$1.5\sigma_{e}^{2}$	0.0570	0.0630	0.0618		0.8878	0.8974	0.9541
Unblinded	$0.5\sigma_{c}^{2}$	$0.5\sigma_{e}^{2}$	0.0629	0.0604	0.0503		0.8693	0.8799	0.8165
Unblinded	$\sigma_{c}^{2}$	$\sigma_{e}^{2}$	0.0599	0.0652	0.0643		0.8840	0.8843	0.8966
Unblinded	$1.5\sigma_{c}^{2}$	$1.5\sigma_{e}^{2}$	0.0594	0.0641	0.0624		0.8866	0.8955	0.9551
Trial Design Setting 2									
Procedure	${\tilde{\sigma }}_{c}^{2}$	${\tilde{\sigma }}_{e}^{2}$	t=3	t=5	t=7		t=3	t=5	t=7
Blinded	$0.5\sigma_{c}^{2}$	$0.5\sigma_{e}^{2}$	0.0263	0.0254	0.0249		0.8140	0.8282	0.8146
Blinded	$\sigma_{c}^{2}$	$\sigma_{e}^{2}$	0.0261	0.0271	0.0266		0.8159	0.8283	0.8271
Blinded	$1.5\sigma_{c}^{2}$	$1.5\sigma_{e}^{2}$	0.0254	0.0269	0.0262		0.8181	0.8287	0.8883
Unblinded	$0.5\sigma_{c}^{2}$	$0.5\sigma_{e}^{2}$	0.0268	0.0270	0.0258		0.8038	0.8002	0.8003
Unblinded	$\sigma_{c}^{2}$	$\sigma_{e}^{2}$	0.0274	0.0268	0.0267		0.8130	0.8059	0.8030
Unblinded	$1.5\sigma_{c}^{2}$	$1.5\sigma_{e}^{2}$	0.0256	0.0262	0.0262		0.8127	0.8095	0.8891

Results are given for Trial Design Settings 1 and 2, for a selection of possible values for the assumed variance parameters, and as a function of the reestimation time point *t*, when $n_{\text{min}}=1$ and $n_{\text{max}}=$ 1,000.

**Table 4:** Median values of the final total required sample size ($\hat{N}$) required by the blinded ($\tau_{*}=0$) and unblinded reestimation procedures are shown

			τ=0				τ=δ		
Trial Design Setting 1									
Procedure	${\tilde{\sigma }}_{c}^{2}$	${\tilde{\sigma }}_{e}^{2}$	t=2	t=3	t=4		t=2	t=3	t=4
Blinded	$0.5\sigma_{c}^{2}$	$0.5\sigma_{e}^{2}$	1,312	1,556	4,560		1,348	1,612	4,560
Blinded	$\sigma_{c}^{2}$	$\sigma_{e}^{2}$	1,352	1,352	1,336		1,376	1,392	1,392
Blinded	$1.5\sigma_{c}^{2}$	$1.5\sigma_{e}^{2}$	1,456	1,388	1,716		1,468	1,420	1,716
Unblinded	$0.5\sigma_{c}^{2}$	$0.5\sigma_{e}^{2}$	1,300	1,556	4,560		1,300	1,556	4,560
Unblinded	$\sigma_{c}^{2}$	$\sigma_{e}^{2}$	1,352	1,352	1,328		1,352	1,352	1,332
Unblinded	$1.5\sigma_{c}^{2}$	$1.5\sigma_{e}^{2}$	1,456	1,388	1,716		1,456	1,388	1,716
Trial Design Setting 2									
Procedure	${\tilde{\sigma }}_{c}^{2}$	${\tilde{\sigma }}_{e}^{2}$	t=3	t=5	t=7		t=3	t=5	t=7
Blinded	$0.5\sigma_{c}^{2}$	$0.5\sigma_{e}^{2}$	1,320	1,440	1,960		1,320	1,600	2,160
Blinded	$\sigma_{c}^{2}$	$\sigma_{e}^{2}$	1,260	1,260	1,260		1,380	1,340	1,380
Blinded	$1.5\sigma_{c}^{2}$	$1.5\sigma_{e}^{2}$	1,380	1,260	1,580		1,380	1,340	1,580
Unblinded	$0.5\sigma_{c}^{2}$	$0.5\sigma_{e}^{2}$	1,320	1,440	2,000		1,320	1,440	2,000
Unblinded	$\sigma_{c}^{2}$	$\sigma_{e}^{2}$	1,260	1,260	1,260		1,260	1,260	1,260
Unblinded	$1.5\sigma_{c}^{2}$	$1.5\sigma_{e}^{2}$	1,380	1,260	1,580		1,380	1,260	1,580

Results are given for Trial Design Settings 1 and 2, for a selection of possible values for the assumed variance parameters, and as a function of the reestimation time point *t*, when $n_{\text{min}}=1$ and $n_{\text{max}}=$ 1,000.

We observe no clear trend to the ETI as *t* is increased in either TDS. In TDS2, the ETI is comparable for each *t*, for each of the different assumed combinations of variance parameters. However, in TDS1, the ETI is more sensitive to the choice of *t*. As in Section 3.1, this is likely a consequence of the smaller number of clusters for this TDS.

In TDS2, each of the values of *t* confers the largest EP for some combination of the variance parameters. While in TDS1, t=3 or t=4 provide the largest power in at least one considered case. Nonetheless, in some instances, it is clear that placing the reestimation point later in to the trial can cause a substantial loss of power. For example, when ${\tilde{\sigma }}_{c}^{2}=0.5\sigma_{c}^{2}$ and ${\tilde{\sigma }}_{e}^{2}=0.5\sigma_{e}^{2}$ in TDS1, the unblinded procedure has an EP of 0.8799 when t=3, but this drops to 0.8165 for t=4. In addition, Table 4 and Supporting Information Figures 9 and 12 provide a clear warning as to the possible issues with utilizing larger values of *t*: when the variance parameters are underspecified this can lead to far larger sample sizes being required, as extremely large values of $n_{\text{final}}$ are required to attempt to attain the desired power.

Finally, examining Supporting Information Figures 7, 8, 11, and 12, we can see that, as would be anticipated, the reestimation procedures are generally able to more accurately estimate the variance components as *t* is increased. The exception to this rule is the blinded procedure when τ=δ. In this case, larger values of *t* increase the median bias in ${\hat{\sigma }}_{c}^{2}$.

### 3.3 Performance for varying $\tau_{*}$

In this section, we consider the effect the value of $\tau_{*}$ has on the blinded re‐estimation procedure. We set t=3 and t=5 for TDS1 and TDS2, respectively, and explore $\left({\tilde{\sigma }}_{c}^{2},{\tilde{\sigma }}_{e}^{2}\right)\in \left\{0.5\sigma_{c}^{2},\sigma_{c}^{2},1.5\sigma_{c}^{2}\right\}\times \left\{0.5\sigma_{e}^{2},\sigma_{e}^{2},1.5\sigma_{e}^{2}\right\}$, with τ=0 and τ=δ, when $n_{\text{min}}=1$ and $n_{\text{max}}=$ 1,000. Two values of $\tau_{*}$ are considered: $\tau_{*}=0$ and $\tau_{*}=\delta$. Our results are presented in Table 5, which contains the ERRs, and in Supporting Information Figures 13– 16, which display the distributions of ${\hat{\sigma }}_{c}^{2}$ and $\hat{N}$.

**Table 5:** Empirical type‐I error rates (τ=0) and power (τ=δ) of the blinded reestimation procedures are shown

		τ=0			τ=δ	
${\tilde{\sigma }}_{c}^{2}$	${\tilde{\sigma }}_{e}^{2}$	$\tau_{*}=0$	$\tau_{*}=\delta$		$\tau_{*}=0$	$\tau_{*}=\delta$
Trial Design Setting 1						
$0.5\sigma_{c}^{2}$	$0.5\sigma_{e}^{2}$	0.0581	0.0581		0.8812	0.8739
$0.5\sigma_{c}^{2}$	$\sigma_{e}^{2}$	0.0589	0.0596		0.8859	0.8779
$0.5\sigma_{c}^{2}$	$1.5\sigma_{e}^{2}$	0.0632	0.0634		0.8935	0.8905
$\sigma_{c}^{2}$	$0.5\sigma_{e}^{2}$	0.0568	0.0573		0.8817	0.8739
$\sigma_{c}^{2}$	$\sigma_{e}^{2}$	0.0593	0.0609		0.8848	0.8798
$\sigma_{c}^{2}$	$1.5\sigma_{e}^{2}$	0.0643	0.0626		0.8957	0.8961
$1.5\sigma_{c}^{2}$	$0.5\sigma_{e}^{2}$	0.0583	0.0563		0.8825	0.8736
$1.5\sigma_{c}^{2}$	$\sigma_{e}^{2}$	0.0594	0.0584		0.8862	0.8801
$1.5\sigma_{c}^{2}$	$1.5\sigma_{e}^{2}$	0.0630	0.0642		0.8974	0.8944
Trial Design Setting 2						
$0.5\sigma_{c}^{2}$	$0.5\sigma_{e}^{2}$	0.0254	0.0262		0.8282	0.7876
$0.5\sigma_{c}^{2}$	$\sigma_{e}^{2}$	0.0260	0.0254		0.8284	0.7996
$0.5\sigma_{c}^{2}$	$1.5\sigma_{e}^{2}$	0.0269	0.0260		0.8284	0.8026
$\sigma_{c}^{2}$	$0.5\sigma_{e}^{2}$	0.0260	0.0263		0.8295	0.7880
$\sigma_{c}^{2}$	$\sigma_{e}^{2}$	0.0271	0.0263		0.8283	0.7995
$\sigma_{c}^{2}$	$1.5\sigma_{e}^{2}$	0.0262	0.0269		0.8301	0.8096
$1.5\sigma_{c}^{2}$	$0.5\sigma_{e}^{2}$	0.0266	0.0258		0.8261	0.7881
$1.5\sigma_{c}^{2}$	$\sigma_{e}^{2}$	0.0258	0.0251		0.8289	0.7991
$1.5\sigma_{c}^{2}$	$1.5\sigma_{e}^{2}$	0.0269	0.0269		0.8287	0.8071

Results are given for Trial Design Settings 1 (t=3) and 2 (t=5), for a selection of possible values for the assumed variance parameters, and as a function of $\tau_{*}$, when $n_{\text{min}}=1$ and $n_{\text{max}}=$ 1,000.

We can see that the value of $\tau_{*}$ appears to have little influence on the ETI for either TDS. However, in some cases the choice of $\tau_{*}$ has a notable effect on the EP, with specifically $\tau_{*}=\delta$ reducing the EP. This is especially evident in TDS2. From Supporting Information Figures 13–16 we can see why this is the case. When $\tau_{*}=\delta$, on average, reduced values for ${\hat{\sigma }}_{c}^{2}$ are obtained compared to choosing $\tau_{*}=0$. Thus $\tau_{*}=0$ results in larger final requisite sample sizes, which provides an increase to the EP.

## 4 DISCUSSION

In this article, we have presented blinded and unblinded SSRE procedures for cross‐sectional SW‐CRTs. These methods should assist with scenarios in which there is difficultly in determining a trial's required sample size because of the need to specify values for several nuisance parameters. We were able to demonstrate that, at least for the considered scenarios, the SSRE procedures could increase power substantially over a conventional SW‐CRT design when the variance parameters were underspecified.

Unfortunately, in TDS1 there were instances of substantial inflation to the ETI rate using our SSRE procedures (Table 1). This was not surprising given the extremely low number of clusters in this scenario, with past research highlighting issues in such settings (Taljaard, Teerenstra, Ivers, & Fergusson, 2016; Grayling et al., 2017b). Additionally, it follows results observed for parallel‐group CRTs (van Schie & Moerbeek, 2014). To address this, one could look to use the Kenward‐Roger correction for performing a hypothesis test on a fixed effect in a linear‐mixed model (Kenward & Roger, 1997). In practice this would be expected to be useful, as it was indeed recently demonstrated to be for parallel group CRTs (Kahan et al., 2016). However, it is extremely computationally intensive to examine the utility of this within the context of a large scale SSRE simulation study. We thus omit such considerations here. Alternatively, an alpha‐level adjustment procedure, as considered for example by Golkowski et al. (2014) and Friede and Stammer (2010), among others, could assist in controlling the type‐I error rate more accurately. This approach seeks to identify the largest possible inflation that could occur, and lowers the value of α used in the final test accordingly to control to the nominal level for this worst case scenario. Of course, when utilizing either of these approaches one must bare in mind that a penalty to the EP would likely be observed.

Moreover, in TDS1 the SSRE procedures routinely did not display an EP above the nominal level (Table 1). To combat this, one could employ a sample size inflation factor, as proposed by Zucker, Wittes, Schabenberger, and Brittan (1999). This has been demonstrated to be highly effective in a range of trial design settings (e.g., Friede & Kieser, 2013; Golkowski et al., 2014).

Nonetheless, it was clear that though the SSRE procedure did not in many instances precisely meet the desired operating characteristics, their performance in comparison to the fixed sample design was often impressive. When the variance parameters were overspecified, setting $n_{\text{min}}<n_{\text{init}}$ allowed the SSRE procedures to reduce the group size and attain a power closer to the nominal level. Similarly statements hold in regard to when the variance parameters were underspecified having set $n_{\text{max}}>n_{\text{init}}$. Thus the SSRE designs may have real utility in combating issues of uncertainty over $\sigma_{c}^{2}$ and $\sigma_{e}^{2}$ at the design stage, especially if an adjustment procedure (like those described above) is incorporated to enhance their ability to attain the desired type‐I and type‐II error rates. Choosing between the blinded and unblinded procedures may be more challenging however. As seen in Table 2, the blinded procedure typically requires a larger sample size for only a small gain in power over an unblinded option. This, in particular, would need to be taken into account to choose the preferred reestimation approach on statistical grounds.

Of course, in practice, there are further important operational details that must be taken into account in order to make a choice between the blinded and unblinded procedures. For example, utilizing unblinded reestimation can induce additional complexities. In particular, an independent data monitoring committee may then be required, in order to ensure that study personnel remain blinded.

In my opinion, it is important to highlight that this is not only a choice between statistical operating characteristics, but also a decision for which operational aspects have to be taken into account. An unblinded sample size reestimation needs careful planning, and potentially an independent data monitoring committee, to assure that the study personnel stays blinded.

From Table 1 and Supporting Information Table 1 in particular, it is clear that altering the value of ${\tilde{\sigma }}_{c}^{2}$, for fixed ${\tilde{\sigma }}_{e}^{2}$, has little effect on the performance of the reestimation procedures. As noted by one of the anonymous reviewers, it is possible, however, that this may not be the case when $\sigma_{c}^{2}\approx \sigma_{e}^{2}$. We explore such scenarios in the Supplementary Material. Ultimately, we find that the performance of the reestimation procedures is insensitive to the values of ${\tilde{\sigma }}_{c}^{2}$ and ${\tilde{\sigma }}_{e}^{2}$ even when $\sigma_{c}^{2}\approx \sigma_{e}^{2}$.

In addition, given the fact that ${\hat{\sigma }}_{c}^{2}$ will likely in general be biased for $\sigma_{c}^{2}$ when using the blinded procedure, it is reasonable to ask how well the blinded procedure could perform if we simply assume that $\sigma_{c}^{2}=0$. We also consider such scenarios in the Supplementary Material. From this, we caution against assuming the between cluster variance is not of importance, as the trial operating characteristics can be severely effected if this is not the case.

We observed that the ETI and EP were similar for several choices of *t*, particularly in TDS1, but the EP was sometimes substantially lower if the reestimation point was late in the trial (Table 3). This should not be surprising. There is clearly a trade‐off to be made in terms of how late in the trial one places the reestimation point. The longer we wait, the more accurately we are in general able to estimate the variance parameters. However, we then have less time to readjust for any pretrial misspecification. Indeed, this point is a complex one, related to recent research on the value of each cluster‐period in a SW‐CRT (Kasza & Forbes, 2017), and how horizontal and vertical components contribute to the treatment effect estimate in a SW‐CRT (Matthews & Forbes, 2017). As an example, consider the choice t=4 in TDS1. This leaves a single time period left in which to carry out the trial with a modified sample size. However, with our choice of *X*, in this final time period all of the clusters receive the experimental intervention. The implication of this is that the majority of the cluster‐periods in time period five actually contribute little to estimating the treatment effect. Consequently, even taking $n_{\text{final}}=$ 1,000 may not allow the desired power to be attained if the preceding time periods were conducted with too small a sample size. Accordingly, one may therefore suggest an intermediate value for *t*, such as t=3 in TDS1, to be preferable in most cases.

From Section S.M.7 in the Supplementary Material, it is clear that when specifying a SSRE procedure, it is important to choose the values of $n_{\text{min}}$ and $n_{\text{max}}$ carefully. In particular, while it may be preferable to have $n_{\text{min}}<n_{\text{init}}$, this could have negative consequences upon the EP. However, this does confer an advantage that when ${\tilde{\sigma }}_{e}^{2}=1.5\sigma_{e}^{2}$ the SSRE procedures were able to reduce the power to closer to the nominal level. Likewise, increasing the value of $n_{\text{max}}$ may seem beneficial, but one then needs both to be able to find more patients to recruit in the later periods, and also to be able to logistically handle a larger sample size. This may be a problem particularly for scenarios where the SW‐CRT design is being utilized because of resource constraints.

Similarly, if using the blinded SSRE procedure, the value for $\tau_{*}$ should be chosen with care. From Table 5, it was evident that this can have consequences on, in particular, the EP. As has been previously noted for conventional parallel arm trials (Kieser & Friede, 2003), the bias introduced into the estimate of $\sigma_{c}^{2}$ when τ=δ, may make the choice $\tau_{*}=0$ preferable. However, one should be careful not to automatically assume this is the case. Specifically, the choice $\tau_{*}=0$ will likely often provide greater power than the choice $\tau_{*}=\delta$. But, in certain cases $\tau_{*}=\delta$ may in its own right provide the desired power, as is approximately the case for TDS2 by Table 5. In this instance, the increased power attained by choosing $\tau_{*}=0$ could be disadvantageous because of the associated increased required sample sizes. As general guidance, one may anticipate that $\tau_{*}=0$ would be preferable for settings with small numbers of clusters, where attaining the desired power may be more challenging, as seen for TDS1.

An additional point of note is our method for specifying ${\hat{\sigma }}_{c}^{2}$ in the blinded re‐estimation procedure. Explicitly, we choose to set ${\hat{\sigma }}_{c}^{2}=f\left({\bar{S}}_{Ct-t}^{2},{\hat{\sigma }}_{e}^{2},X^{\left(t\right)},n_{\text{init}},0\right)$ when $f\left({\bar{S}}_{Ct-t}^{2},{\hat{\sigma }}_{e}^{2},X^{\left(t\right)},n_{\text{init}},\tau_{*}\right)<0<f\left({\bar{S}}_{Ct-t}^{2},{\hat{\sigma }}_{e}^{2},X^{\left(t\right)},n_{\text{init}},0\right)$. It may appear more logical to set ${\hat{\sigma }}_{c}^{2}=0$ in this instance. We made our choice based on the fact that it would be rare for one to truly believe that $\sigma_{c}^{2}=0$. Consequently, $f({\bar{S}}_{Ct-t}^{2},{\hat{\sigma }}_{e}^{2},X^{\left(t\right)},n_{\text{init}},\tau_{*})<0$ we felt would more likely indicate that an overly large value for $\tau_{*}$ had been chosen. In practice though, this specification would likely have little influence on the performance of the blinded reestimation procedure unless the magnitude of $\tau -\tau_{*}$ is large.

Note also that throughout this work we considered only cases with π=0. However, the SSRE procedures considered here are only asymptotically invariant to the value of the period effects. It would thus in general be important to assess the effect of nonzero period effects on the SSRE procedures, though it is reasonable to anticipate that it will be small.

There are several practical factors that must be considered before SSRE is incorporated into a SW‐CRT design. Primarily, our methodology is dependent upon data from all clusters being available for analysis immediately following period *t*. The efficiency of the procedures would suffer if this were not the case. Therefore, it would be important for measures to be put in place for efficient data collection, storage, and analysis. In addition, there may be some instances where SSRE is not realistic. For example, if the intervention was a planned roll‐out that is part of a larger programme implementation. A trialist must consider their scenario carefully before utilizing SSRE.

Several possible extensions to our procedures are possible. Firstly, we here only addressed cross‐sectional SW‐CRT designs analyzed with the Hussey and Hughes model. Though the majority of SW‐CRT research has been set in this domain, it would be beneficial to also establish methods to incorporate SSRE in to cohort designed SW‐CRT, different endpoints of interest, or indeed different analysis models. While it would be relatively simple to explore the performance of an unblinded procedure in these settings, methodology for blinded reestimation would be more complex. Similar statements also hold for allowing variable cluster sizes, and also incorporating the interim estimated value for τ in to the re‐estimation procedure.

Additionally, we considered here a scenario in which the number of clusters remained fixed throughout the trial; adjusting only the per cluster per period sample size following the reestimation point. One could also explore the performance of a procedure that increases the value of *C* following reestimation, creating an incomplete‐block SW‐CRT. For scenarios in which patients are hard to come by, but clusters are not, this would be a useful extension.

It is worth noting that our procedures are actually applicable to any cross‐sectional CRT design to be analyzed with the Hussey and Hughes model. This means, for example, that it would allow also the incorporation of SSRE in to a cluster randomised crossover trial, which is being increasingly acknowledged in the trials community as a useful design (Arnup et al., 2014).

Regardless of the practical considerations discussed above, and the possible future avenues of extension to our methods, it is clear that the ability to include a SSRE point in to SW‐CRT designs is a useful addition to the methodologists toolbox.

## CONFLICT OF INTEREST

The authors have declared no conflict of interest.

## Supporting information

Supplementary Material

Supplementary Material
