# Supplementary material for: Blinded and unblinded sample size reestimation procedures for stepped‐wedge cluster randomized trials
Source: Biom J. 2018 Aug 3;60(5):903–16. doi: 10.1002/bimj.201700125 (PMC6175439; doi:10.1002/bimj.201700125)
Supplement: Supplementary file 2 — Supplementary Material [file BIMJ-60-903-s002.pdf]

Supplementary Material to "Blinded and unblinded sample size re-estimation procedures for stepped-wedge cluster randomized trials"

S.M.1 Expected value of blinded estimators

In this section, we derive the expected value of the blinded estimators given in Section 2.2.

To begin, we observe that for equation (1), for $i_1, i_2 \in \{1, \dots, C\}$, $j_1, j_2 \in \{1, \dots, T\}$, and $k_1, k_2 \in \{1, \dots, n\}$

$$\text{Cov}(Y_{i_1 j_1 k_1}, Y_{i_2 j_2 k_2}) = \delta_{i_1 i_2}\delta_{j_1 j_2}\delta_{k_1 k_2}\sigma_e^2 + \delta_{i_1 i_2}\sigma_c^2,$$

as is stated in Hussey and Hughes [1]. Moreover, using the standard "." notation to indicate when a variable has been summed over, and setting $\mathbb{S}_a = \{(x, y) \in (1, \dots, a) \times (1, \dots, a) : x \neq y\}$, we have

$$\begin{aligned}
\text{Cov}\left(\bar{Y}_{ij.}, \bar{Y}_{ij.}\right) &= \text{Var}\left(\bar{Y}_{ij.}\right), \\
&= \text{Var}\left(\frac{1}{n}\sum_{k=1}^{n} Y_{ijk}\right), \\
&= \frac{1}{n^2}\text{Var}\left(\sum_{k=1}^{n} Y_{ijk}\right), \\
&= \frac{1}{n^2}\left\{\sum_{k=1}^{n}\text{Var}(Y_{ijk}) + \sum_{(k_1,k_2)\in\mathbb{S}_n}\text{Cov}\left(Y_{ijk_1}, Y_{ijk_2}\right)\right\}, \\
&= \frac{1}{n^2}\left\{\sum_{k=1}^{n}(\sigma_e^2 + \sigma_c^2) + \sum_{(k_1,k_2)\in\mathbb{S}_n}\sigma_c^2\right\}, \\
&= \frac{1}{n^2}\left\{n(\sigma_e^2 + \sigma_c^2) + n(n-1)\sigma_c^2\right\}, \\
&= \frac{1}{n}\left(\sigma_e^2 + n\sigma_c^2\right).
\end{aligned}$$

A straight forward adaptation of the above also gives that

$$\text{Cov}\left(\bar{Y}_{.j.}, \bar{Y}_{.j.}\right) = \frac{1}{nC}(\sigma_e^2 + n\sigma_c^2).$$

Now, after time period t, we compute what we refer to as $\bar{S}^2_{Ct-t}$ and S^2_{Ct}, which are given by

$$
\begin{aligned}
\left(\frac{Ct-t}{n}\right)\bar{S}^2_{Ct-t} &= \sum_{i=1}^{C}\sum_{j=1}^{t}\left(\bar{Y}_{ij.}-\bar{Y}_{.j.}\right)^2, \\
&= \sum_{j=1}^{t}\left(\sum_{i=1}^{C}\bar{Y}^2_{ij.}-C\bar{Y}^2_{.j.}\right), \\
(nCt-Ct)S^2_{Ct} &= \sum_{i=1}^{C}\sum_{j=1}^{t}\sum_{k=1}^{n}\left(Y_{ijk}-\bar{Y}_{ij.}\right)^2, \\
&= \sum_{i=1}^{C}\sum_{j=1}^{t}\left(\sum_{k=1}^{n}Y^2_{ijk}-n\bar{Y}^2_{ij.}\right).
\end{aligned}
$$

Without loss of generality, we can assume that $\mu = 0$. By definition, we know that

$$
\mathrm{E}\left\{(nCt-Ct)S^2_{Ct}\right\} = (nCt-Ct)\sigma^2_e.
$$

Furthermore, exploiting the fact that $X^2_{ij} = X_{ij}$

$$
\begin{aligned}
\mathrm{E}\left\{\left(\frac{Ct-t}{n}\right)\bar{S}^2_{Ct-t}\right\} &= \sum_{j=1}^{t}\left\{\sum_{i=1}^{C}\mathrm{E}(\bar{Y}^2_{ij.})-C\mathrm{E}(\bar{Y}^2_{.j.})\right\}, \\
&= \sum_{j=1}^{t}\left[\sum_{i=1}^{C}\left\{\mathrm{Var}(\bar{Y}_{ij.})+\mathrm{E}(\bar{Y}_{ij.})^2\right\}-C\left\{\mathrm{Var}(\bar{Y}_{.j.})+\mathrm{E}(\bar{Y}_{.j.})^2\right\}\right], \\
&= \sum_{j=1}^{t}\left(\sum_{i=1}^{C}\left[\frac{1}{n}(\sigma^2_e+n\sigma^2_c)+\left\{\frac{1}{n}\sum_{k=1}^{n}(\pi_j+X_{ij}\tau)\right\}^2\right]\right. \\
&\qquad \left. -C\left[\frac{1}{nC}(\sigma^2_e+n\sigma^2_c)+\left\{\frac{1}{nC}\sum_{i=1}^{C}\sum_{k=1}^{n}(\pi_j+X_{ij}\tau)\right\}^2\right]\right), \\
&= \frac{Ct}{n}(\sigma^2_e+n\sigma^2_c)-\frac{t}{n}(\sigma^2_e+n\sigma^2_c) \\
&\qquad +\sum_{j=1}^{t}\left[\sum_{i=1}^{C}(\pi_j+X_{ij}\tau)^2-\frac{1}{C}\left\{\sum_{i=1}^{C}(\pi_j+X_{ij}\tau)\right\}^2\right], \\
&= \left(\frac{Ct-t}{n}\right)\sigma^2_e+(Ct-t)\sigma^2_c+\sum_{j=1}^{t}\left[\sum_{i=1}^{C}(\pi^2_j+2\pi_jX_{ij}\tau+X^2_{ij}\tau^2)\right. \\
&\qquad \left. -\frac{1}{C}\left\{\left(\sum_{i=1}^{C}\pi_j\right)^2+2\left(\sum_{i=1}^{C}\pi_j\right)\left(\sum_{i=1}^{C}X_{ij}\tau\right)+\left(\sum_{i=1}^{C}X_{ij}\tau\right)^2\right\}\right], \\
&= \left(\frac{Ct-t}{n}\right)\sigma^2_e+(Ct-t)\sigma^2_c+\sum_{j=1}^{t}\left[C\pi^2_j+2\tau\sum_{i=1}^{C}\pi_jX_{ij}+\tau^2\sum_{i=1}^{C}X_{ij}\right. \\
&\qquad \left. -\frac{1}{C}\left\{C^2\pi^2_j+2C\tau\sum_{i=1}^{C}\pi_jX_{ij}+\tau^2\left(\sum_{i=1}^{C}X_{ij}\right)^2\right\}\right], \\
&= \left(\frac{Ct-t}{n}\right)\sigma^2_e+(Ct-t)\sigma^2_c+\tau^2\sum_{j=1}^{t}\sum_{i=1}^{C}X_{ij}-\frac{\tau^2}{C}\sum_{j=1}^{t}\left(\sum_{i=1}^{C}X_{ij}\right)^2,
\end{aligned}
$$

$$= \left(\frac{Ct-t}{n}\right)\sigma_e^2 + (Ct-t)\sigma_c^2 + \tau^2 \mathbf{1}_t^\top \boldsymbol{X}^{(t)} \mathbf{1}_t - \frac{\tau^2}{C}(\mathbf{1}_t^\top \boldsymbol{X}^{(t)}) \cdot (\mathbf{1}_t^\top \boldsymbol{X}^{(t)}).$$

Therefore, we have

$$\begin{aligned}
\mathrm{E}(\bar{S}_{Ct-t}^2) &= \sigma_e^2 + n\sigma_c^2 + \frac{n\tau^2}{Ct-t}\mathbf{1}_t^\top \boldsymbol{X}^{(t)} \mathbf{1}_t - \frac{n\tau^2}{C(Ct-t)}(\mathbf{1}_t^\top \boldsymbol{X}^{(t)}) \cdot (\mathbf{1}_t^\top \boldsymbol{X}^{(t)}),\\
\mathrm{E}(S_{Ct}^2) &= \sigma_e^2,
\end{aligned}$$

as given in the main part of the paper.

S.M.2 Possible blinded estimators

In our described blinded SSRE procedure, we make use of the variables $\bar{S}_{Ct-t}^2$ and S_{Ct}^2 to re-estimate σ_c^2 and σ_e^2. It is important to realise though that this is not a unique method through which these variance parameters can be re-estimated. Specifically, define

$$\begin{aligned}
S_1^2 &= \frac{1}{nCt-C}\sum_{i=1}^{C}\sum_{j=1}^{t}\sum_{k=1}^{n}(Y_{ijk} - \bar{Y}_{...})^2,\\
S_C^2 &= \frac{1}{nCt-C}\sum_{i=1}^{C}\sum_{j=1}^{t}\sum_{k=1}^{n}(Y_{ijk} - \bar{Y}_{i..})^2,\\
S_t^2 &= \frac{1}{nCt-t}\sum_{i=1}^{C}\sum_{j=1}^{t}\sum_{k=1}^{n}(Y_{ijk} - \bar{Y}_{.j.})^2,\\
\bar{S}_{Ct-C}^2 &= \frac{n}{Ct-C}\sum_{i=1}^{C}\sum_{j=1}^{t}(\bar{Y}_{ij.} - \bar{Y}_{i..})^2,\\
\bar{S}_{Ct-1}^2 &= \frac{n}{Ct-1}\sum_{i=1}^{C}\sum_{j=1}^{t}(\bar{Y}_{ij.} - \bar{Y}_{...})^2,\\
\bar{S}_{C-1}^2 &= \frac{nt}{C-1}\sum_{i=1}^{C}(\bar{Y}_{i..} - \bar{Y}_{...})^2,\\
\bar{S}_{t-1}^2 &= \frac{nC}{t-1}\sum_{j=1}^{t}(\bar{Y}_{.j.} - \bar{Y}_{...})^2.
\end{aligned}$$

By modifying the derivations in Section S.M.1, it can be shown that in the absence of period and treatment effects, we have

$$\begin{aligned}
\mathrm{E}(S_1^2) &= \sigma_e^2 + \left(\frac{nCt-nt}{nCt-1}\right)\sigma_c^2,\\
\mathrm{E}(S_C^2) &= \sigma_e^2,\\
\mathrm{E}(S_t^2) &= \sigma_e^2 + \left(\frac{nCt-nt}{nCt-t}\right)\sigma_c^2,
\end{aligned}$$

$$\begin{aligned}
\mathrm{E}(\bar{S}^2_{Ct-C}) &= \sigma_e^2,\\
\mathrm{E}(\bar{S}^2_{Ct-1}) &= \sigma_e^2 + \left(\frac{nCt-nt}{Ct-1}\right)\sigma_c^2,\\
\mathrm{E}(\bar{S}^2_{C-1}) &= \sigma_e^2 + nt\sigma_c^2,\\
\mathrm{E}(\bar{S}^2_{t-1}) &= \sigma_e^2.
\end{aligned}$$

Consequently, there are four logical variables that could be used to estimate σ_e^2: S^2_{Ct}, S^2_C, $\bar{S}^2_{Ct-C}$, and $\bar{S}^2_{t-1}$. However, it can be shown (by adapting the calculations of the previous section) that only S^2_{Ct} provides an unbiased estimate of σ_e^2 in the presence of non-zero period and treatment effects. Accordingly, it is the most logical variable to use to estimate the residual variance.

Things are slightly more complicated for re-estimating σ_c^2, however. For this, we have five possible variables that could be employed: S_1^2, S_t^2, $\bar{S}^2_{Ct-t}$, $\bar{S}^2_{Ct-1}$, and $\bar{S}^2_{C-1}$. Again, modifying our derivations from Section S.M.1, we can demonstrate that all five variables lead in general to a biased estimate of σ_c^2 in the presence of a non-zero treatment effect. However, only $\mathrm{E}(S_t^2)$ and $\mathrm{E}(\bar{S}^2_{Ct-t})$ are not dependent on the value of the period effects. Thus, it is sensible in general to utilise either S_t^2 or $\bar{S}^2_{Ct-t}$ for re-estimating the between cluster variation. Simple algebraic manipulation reveals that these estimators will in fact lead to the same value for $\hat{\sigma}_c^2$. Consequently, we may use either in practice.

S.M.3 Sample size re-estimation procedures: algorithm

Here, we provide a complete point-by-point algorithm for how the blinded and unblinded re-estimation procedures should be conducted.

Firstly, our blinded SSRE procedure is as follows

1. Specify values for $\boldsymbol{X}$, α, β, δ, $\tilde{\sigma}_c^2$, $\tilde{\sigma}_e^2$, t, τ_*, n_{min} and n_{max}.

2. Perform an initial sample size determination, to acquire n_{init}, assuming $\sigma_e^2 = \tilde{\sigma}_e^2$ and $\sigma_c^2 = \tilde{\sigma}_c^2$.

3. Conduct the trial up to the end of time period t, recruiting n_{init} individuals per cluster per period.

4. Compute $\bar{S}^2_{Ct-t}$ and S^2_{Ct}.

5. Set $\hat{\sigma}_e^2 = S^2_{Ct}$ and

$$\hat{\sigma}_c^2 = \begin{cases} f(\bar{S}^2_{Ct-t}, \hat{\sigma}_e^2, \boldsymbol{X}^{(t)}, n_{\text{init}}, \tau_*) & : \text{if } f(\bar{S}^2_{Ct-t}, \hat{\sigma}_e^2, \boldsymbol{X}^{(t)}, n_{\text{init}}, \tau_*) > 0,\\ f(\bar{S}^2_{Ct-t}, \hat{\sigma}_e^2, \boldsymbol{X}^{(t)}, n_{\text{init}}, 0) & : \text{if } f(\bar{S}^2_{Ct-t}, \hat{\sigma}_e^2, \boldsymbol{X}^{(t)}, n_{\text{init}}, \tau_*) < 0 < f(\bar{S}^2_{Ct-t}, \hat{\sigma}_e^2, \boldsymbol{X}^{(t)}, n_{\text{init}}, 0),\\ 0 & : \text{otherwise.} \end{cases}$$

Note: this is the specification of $\hat{\sigma}_c^2$ when we do not assume $\sigma_c^2 = 0$, as in Section S.M.8.

6. Compute the exact required per cluster per period sample size, n_{reest}, for the rest of the trial to imply the desired operating characteristics, assuming $\sigma_c^2 = \hat{\sigma}_c^2$ and $\sigma_e^2 = \hat{\sigma}_e^2$. Then, set n_{final} as follows

$$n_{\text{final}} = \begin{cases} n_{\text{min}} & : \text{if } n_{\text{reest}} < n_{\text{min}}, \\ n_{\text{reest}} & : \text{if } n_{\text{min}} \leq n_{\text{reest}} \leq n_{\text{max}}, \\ n_{\text{max}} & : \text{if } n_{\text{max}} < n_{\text{reest}}. \end{cases}$$

7. Conduct periods $t+1, \dots, T$ of the trial, recruiting n_{final} patients per cluster per period.

8. Perform a final unblinded analysis on all accumulated data using equation (1) to determine efficacy.

Additionally, our unblinded SSRE procedure is as follows

1. Specify values for $\boldsymbol{X}$, α, β, δ, $\tilde{\sigma}_c^2$, $\tilde{\sigma}_e^2$, t, n_{min} and n_{max}.

2. Perform an initial sample size determination, to acquire n_{init}, assuming $\sigma_e^2 = \tilde{\sigma}_e^2$ and $\sigma_c^2 = \tilde{\sigma}_c^2$.

3. Conduct the trial up to the end of time period t, recruiting n_{init} individuals per cluster per period.

4. Fit the following model to all accumulated data using REML estimation

$$Y_{ijk} = \begin{cases} \mu + c_i + \pi_j + X_{ij}\tau + \epsilon_{ijk} & : \text{if } \mathbf{1}_t^\top \boldsymbol{X}^{(t)} \mathbf{1}_t > 0 \text{ and } t > 1, \\ \mu + c_i + \pi_j + \epsilon_{ijk} & : \text{if } \mathbf{1}_t^\top \boldsymbol{X}^{(t)} \mathbf{1}_t = 0 \text{ and } t > 1, \\ \mu + c_i + X_{ij}\tau + \epsilon_{ijk} & : \text{if } \mathbf{1}_t^\top \boldsymbol{X}^{(t)} \mathbf{1}_t > 0 \text{ and } t = 1, \\ \mu + c_i + \epsilon_{ijk} & : \text{if } \mathbf{1}_t^\top \boldsymbol{X}^{(t)} \mathbf{1}_t = 0 \text{ and } t = 1. \end{cases}.$$

5. From the fitted model obtain the estimates $\hat{\sigma}_c^2$ and $\hat{\sigma}_e^2$.

6. Compute the exact required per cluster per period sample size, n_{reest}, for the rest of the trial to imply the desired operating characteristics assuming $\sigma_c^2 = \hat{\sigma}_c^2$ and $\sigma_e^2 = \hat{\sigma}_e^2$. Then, set n_{final} as follows

$$n_{\text{final}} = \begin{cases} n_{\text{min}} & : \text{if } n_{\text{reest}} < n_{\text{min}}, \\ n_{\text{reest}} & : \text{if } n_{\text{min}} \leq n_{\text{reest}} \leq n_{\text{max}}, \\ n_{\text{max}} & : \text{if } n_{\text{max}} < n_{\text{reest}}. \end{cases}$$

7. Conduct periods $t+1, \dots, T$ of the trial, recruiting n_{final} patients per cluster per period.

8. Perform a final unblinded analysis on all accumulated data using equation (1) to determine efficacy.

S.M.4 Performance for varying $\tilde{\sigma}_c^2$ and $\tilde{\sigma}_e^2$

In this section, we provide figures depicting the distributions of $\hat{\sigma}_c^2$, $\hat{\sigma}_e^2$, and $\hat{N}$, corresponding to the design scenarios discussed in Section 3.1 (Supplementary Figures 1-6). From Supplementary Figures 1-2 and 4-5, we can see that the blinded and unblinded procedures provide similar distributions for $\hat{\sigma}_e^2$ when $\tau = 0$ or $\tau = \delta$. The same is also true of $\hat{\sigma}_c^2$ when $\tau = 0$. However, when $\tau = \delta$, the unblinded procedure tends to under-estimate σ_c^2, and the blinded procedure over-estimate σ_c^2. This has implications that can be observed in Supplementary Figures 3 and 6, in that, $\hat{N}$ is generally similar for the blinded and unblinded procedures for $\tau = 0$ (where the distributions for the blinded procedures are shifted up slightly compared to their unblinded counterparts), but not for $\tau = \delta$.

S.M.5 Performance for varying t

Here, we provide figures depicting the distributions of $\hat{\sigma}_c^2$, $\hat{\sigma}_e^2$, and $\hat{N}$, corresponding to the design scenarios discussed in Section 3.2 (Supplementary Figures 7-12). As noted in the main manuscript, increasing the value of t generally leads to minor improvements in the median estimates of $\hat{\sigma}_c^2$ and $\hat{\sigma}_e^2$, and also reduces the variance in the estimates. As can be seen from Supplementary Figures 8 and 11, the exception to this rule is the blinded procedure when $\tau = \delta$, where increasing t can lead to increased bias in the estimates of $\hat{\sigma}_c^2$. The distributions of $\hat{N}$, seen in Supplementary Figures 9 and 12, are as would be expected based on Supplementary Figures 7-8 and 10-11.

S.M.6 Performance for varying τ_*

Next, we provide figures depicting the distributions of $\hat{\sigma}_c^2$ and $\hat{N}$, corresponding to the design scenarios discussed in Section 3.3 (Supplementary Figures 13-16). We can see that the value of τ_* has a strong influence on the distribution of $\hat{\sigma}_c^2$, particularly when $\tau = \delta$. This has implications for the distribution of $\hat{N}$ (Supplementary Figures 14 and 16). Precisely, as noted in the main manuscript, the larger values of $\hat{\sigma}_c^2$ provided by the choice $\tau_* = 0$ when $\tau = \delta$ tends to increase $\hat{N}$, which in turn increases the EP of this approach compared to taking $\tau_* = \delta$.

S.M.7 Performance for varying n_{min} and n_{max}

We now consider how the SSRE procedures perform for different possible combinations of n_{min} and n_{max}. Having already considered the case with $n_{\text{min}} = 1$ and $n_{\text{max}} = 1000$ in Section 3.1, we now examine the other two possible combinations of n_{min} and n_{max} listed in Section 2.3. As in Section 3.1, we set $t = 3$ and $t = 5$ for TDS1 and TDS2 respectively, and explore $(\tilde{\sigma}_c^2, \tilde{\sigma}_e^2) \in \{0.5\sigma_c^2, \sigma_c^2, 1.5\sigma_c^2\} \times \{0.5\sigma_e^2, \sigma_e^2, 1.5\sigma_e^2\}$, with $\tau = 0$ or $\tau = \delta$. For the blinded procedure, we take $\tau_* = 0$. Our results are provided in Supplementary Table 1, which contains the ERRs,

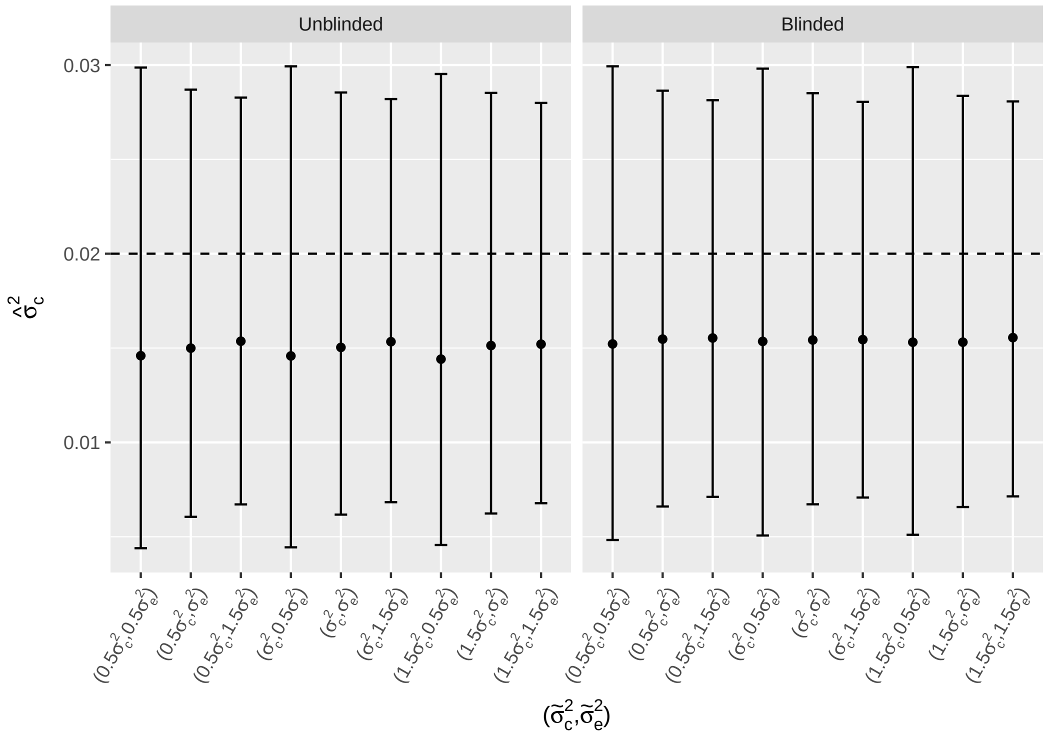

(a) $\hat{\sigma}_c^2$

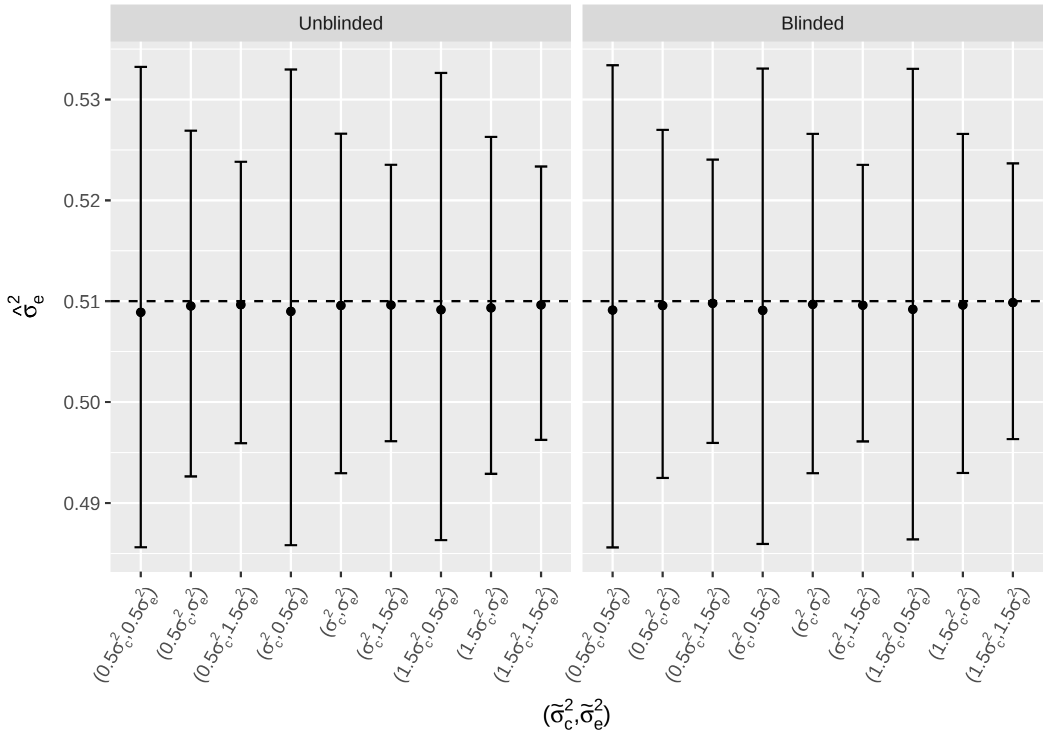

(b) $\hat{\sigma}_e^2$

Supplementary Figure 1: Distributions of $\hat{\sigma}_c^2$ and $\hat{\sigma}_e^2$ are shown via their 25th, 50th and 75th percentiles across the replicate simulations. Results are presented for scenarios with $\tau = 0$, using the blinded ($\tau_* = 0$) and unblinded re-estimation procedures. They are given for Trial Design Setting 1 ($t = 3$), for a selection of possible values for the assumed variance parameters, with $n_{\text{min}} = 1$ and $n_{\text{max}} = 1000$.

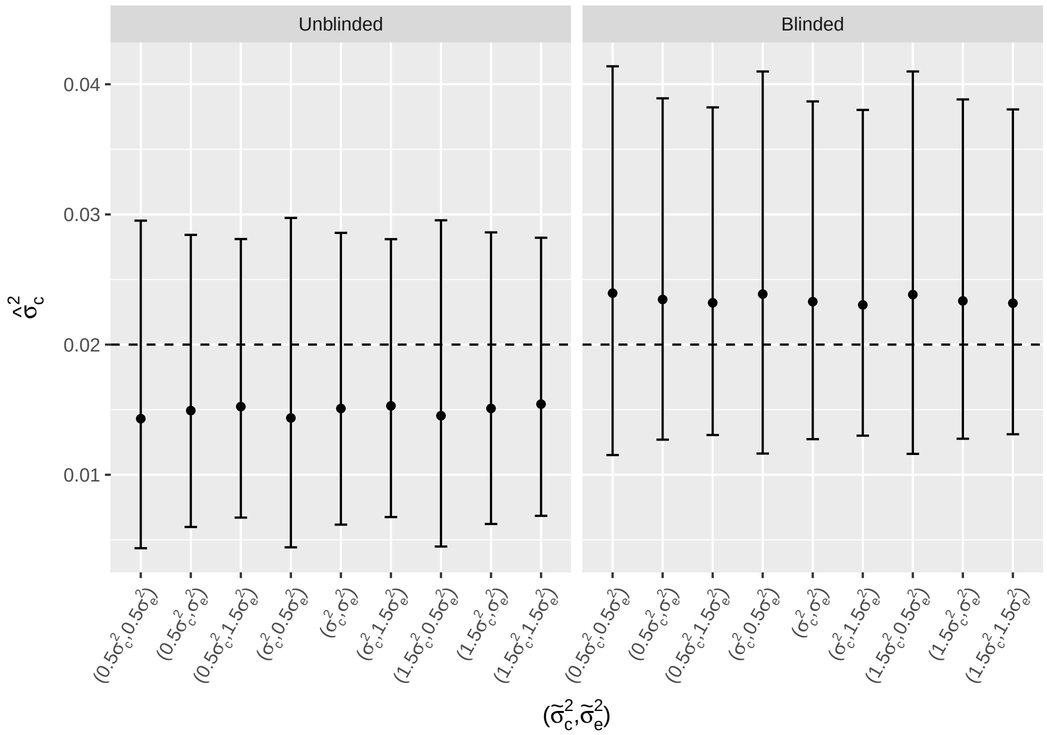

(a) $\hat{\sigma}_c^2$

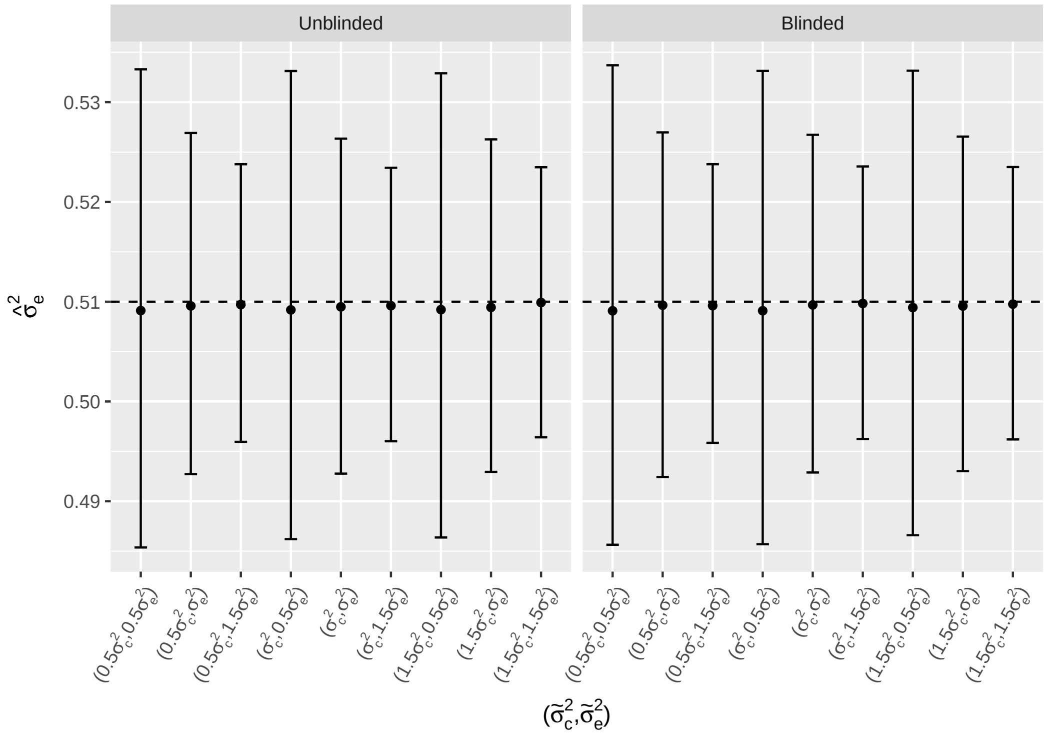

(b) $\hat{\sigma}_e^2$

Supplementary Figure 2: Distributions of $\hat{\sigma}_c^2$ and $\hat{\sigma}_e^2$ are shown via their 25th, 50th and 75th percentiles across the replicate simulations. Results are presented for scenarios with $\tau = \delta$, using the blinded ($\tau_* = 0$) and unblinded re-estimation procedures. They are given for Trial Design Setting 1 ($t = 3$), for a selection of possible values for the assumed variance parameters, with $n_{\text{min}} = 1$ and $n_{\text{max}} = 1000$.

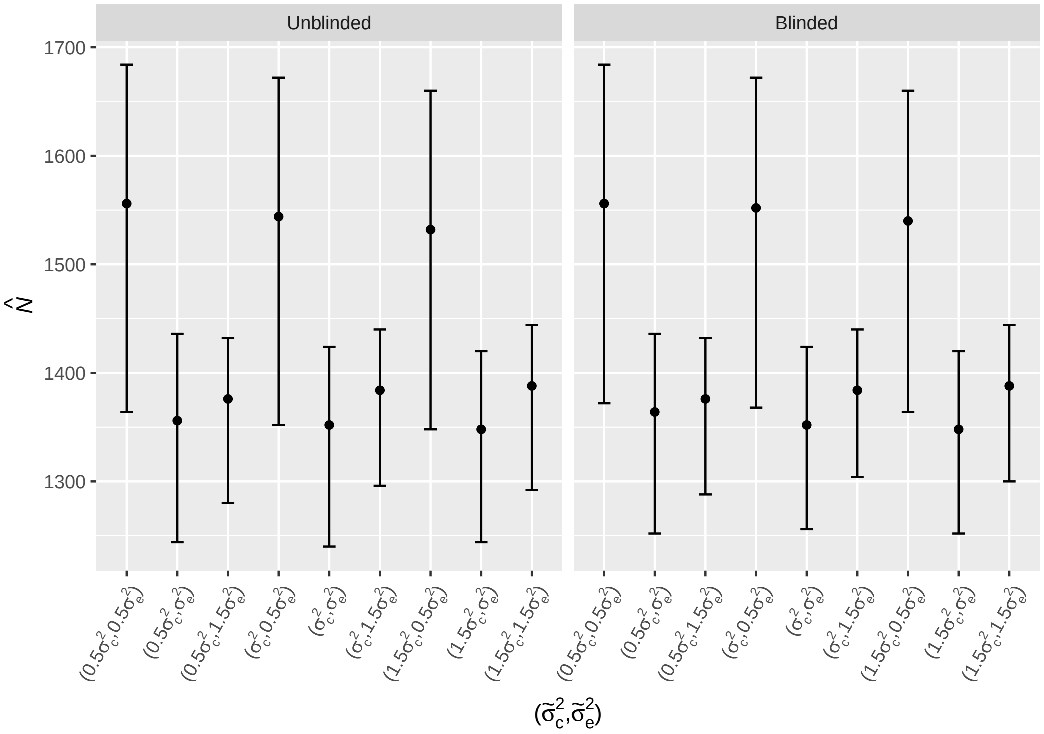

(a) $\tau = 0$

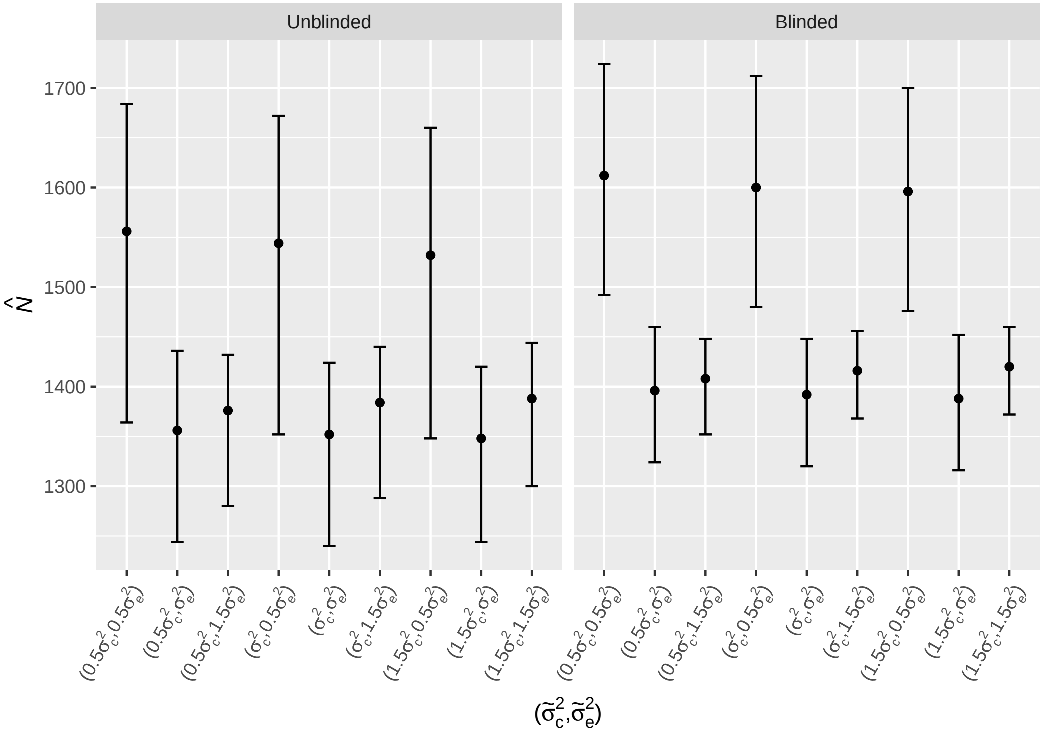

(b) $\tau = \delta$

Supplementary Figure 3: Distributions of $\hat{N}$ are shown via their 25th, 50th and 75th percentiles across the replicate simulations. Results are presented for scenarios with $\tau = 0$ and $\tau = \delta$, using the blinded ($\tau_* = 0$) and unblinded re-estimation procedures. They are given for Trial Design Setting 1 ($t = 3$), for a selection of possible values for the assumed variance parameters, with $n_{\text{min}} = 1$ and $n_{\text{max}} = 1000$.

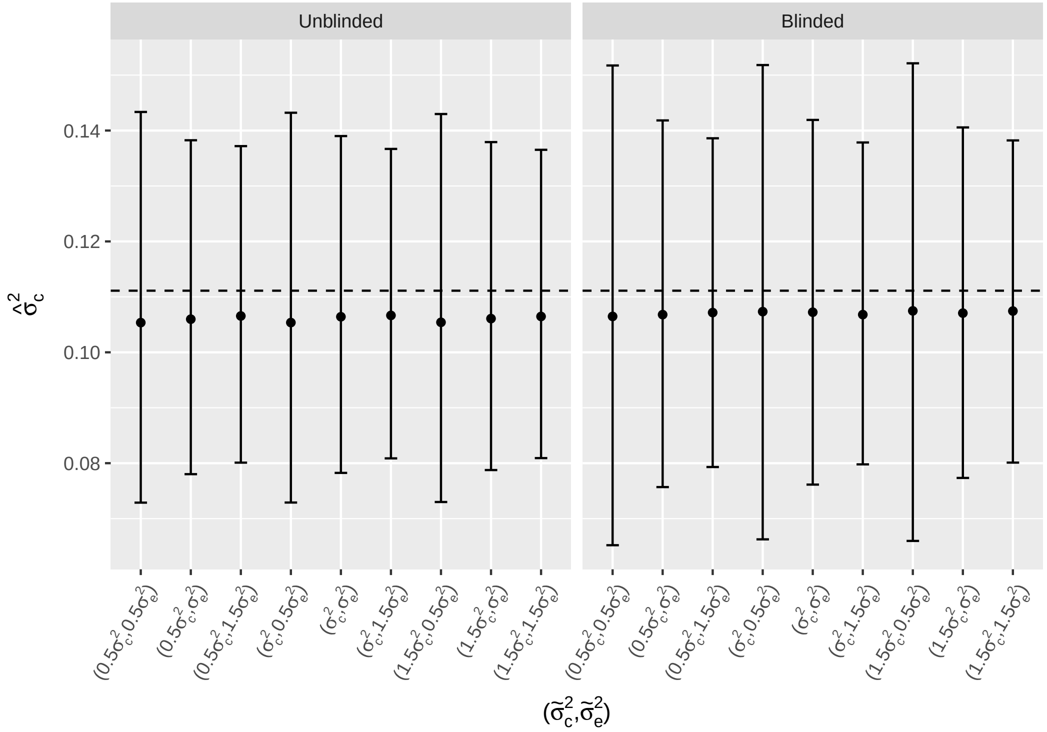

(a) $\hat{\sigma}_c^2$

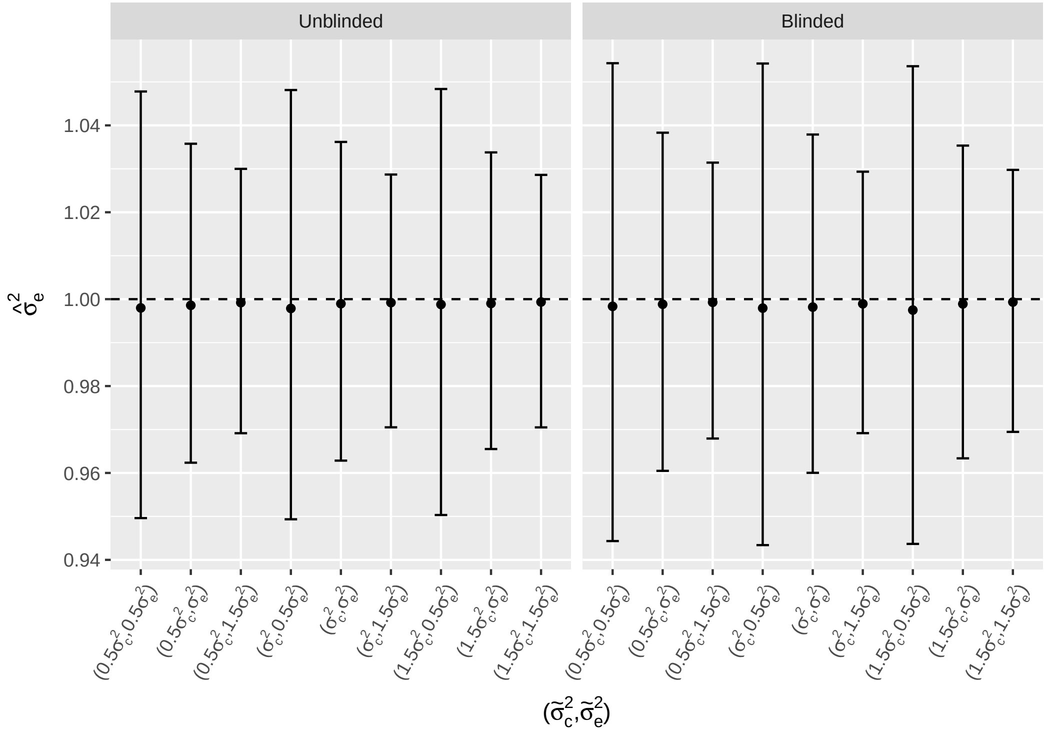

(b) $\hat{\sigma}_e^2$

Supplementary Figure 4: Distributions of $\hat{\sigma}_c^2$ and $\hat{\sigma}_e^2$ are shown via their 25th, 50th and 75th percentiles across the replicate simulations. Results are presented for scenarios with $\tau = 0$, using the blinded ($\tau_* = 0$) and unblinded re-estimation procedures. They are given for Trial Design Setting 2 ($t = 5$), for a selection of possible values for the assumed variance parameters, with $n_{\text{min}} = 1$ and $n_{\text{max}} = 1000$.

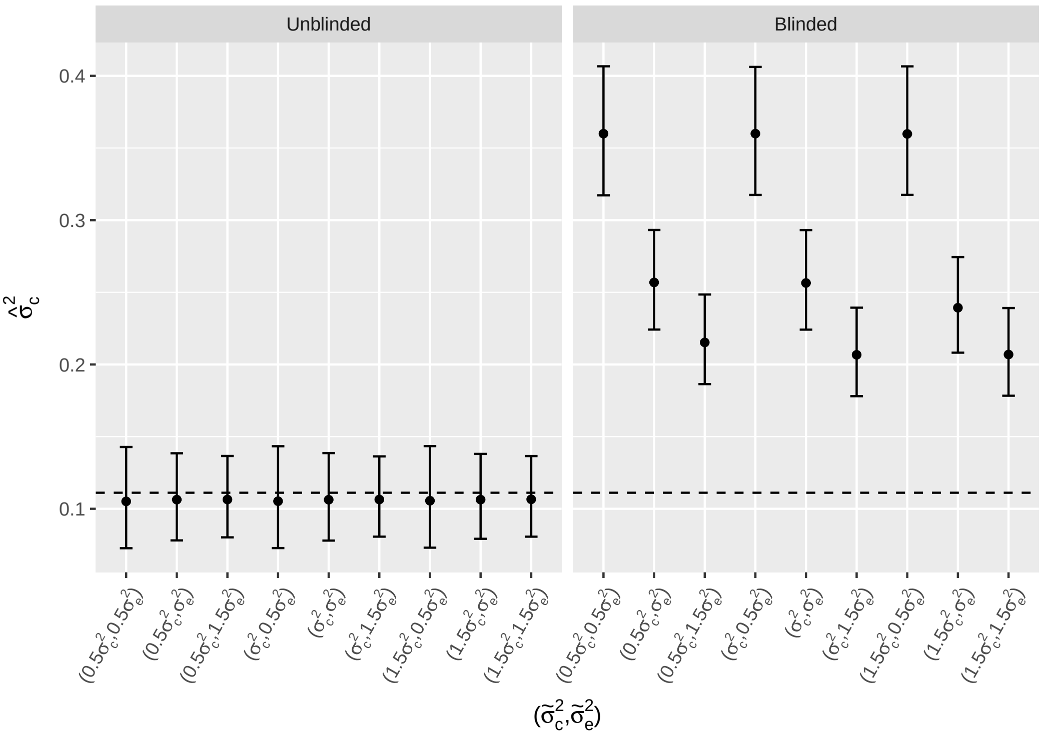

(a) $\hat{\sigma}_c^2$

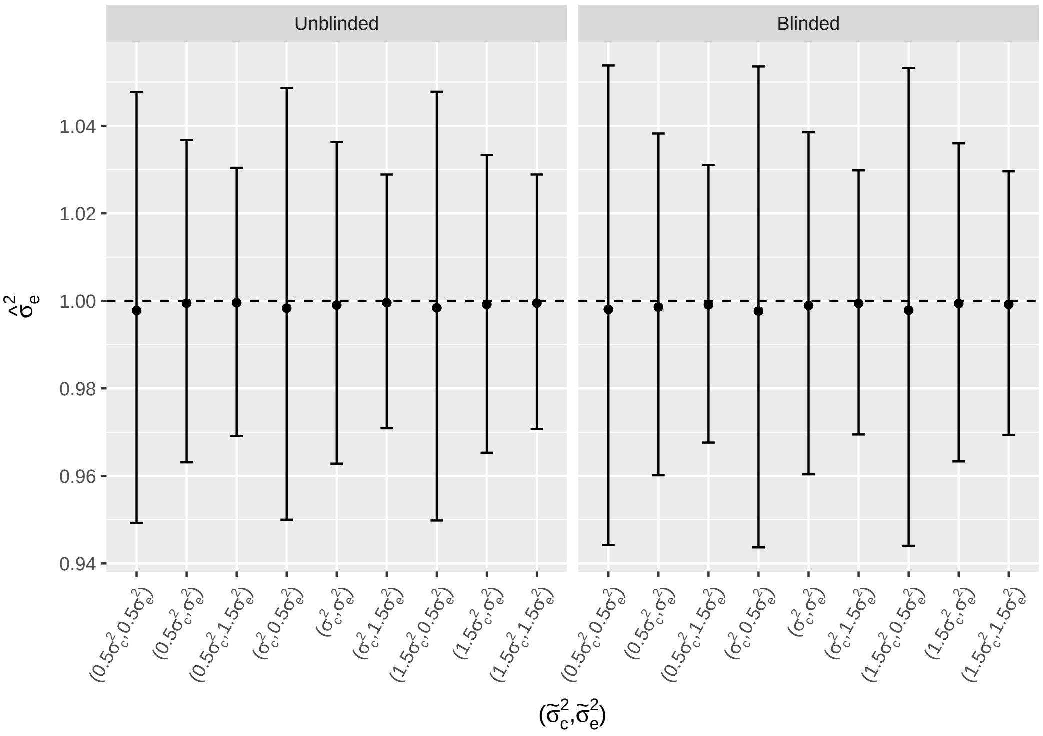

(b) $\hat{\sigma}_e^2$

Supplementary Figure 5: Distributions of $\hat{\sigma}_c^2$ and $\hat{\sigma}_e^2$ are shown via their 25th, 50th and 75th percentiles across the replicate simulations. Results are presented for scenarios with $\tau = \delta$, using the blinded ($\tau_* = 0$) and unblinded re-estimation procedures. They are given for Trial Design Setting 2 ($t = 5$), for a selection of possible values for the assumed variance parameters, with $n_{\text{min}} = 1$ and $n_{\text{max}} = 1000$.

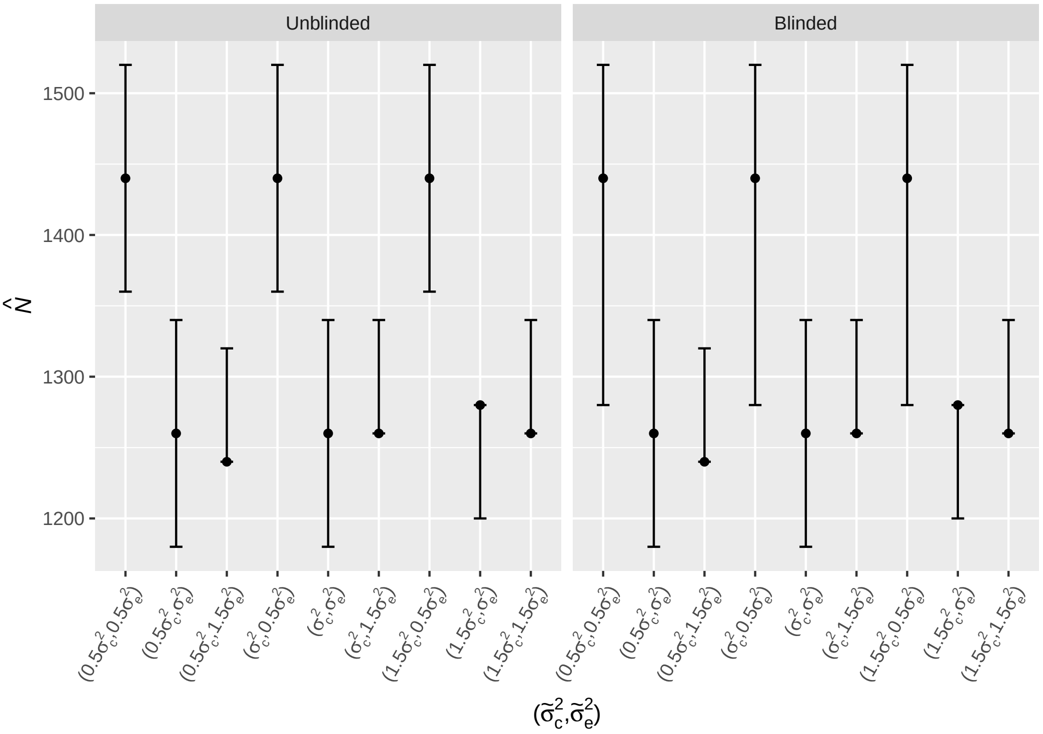

(a) $\tau = 0$

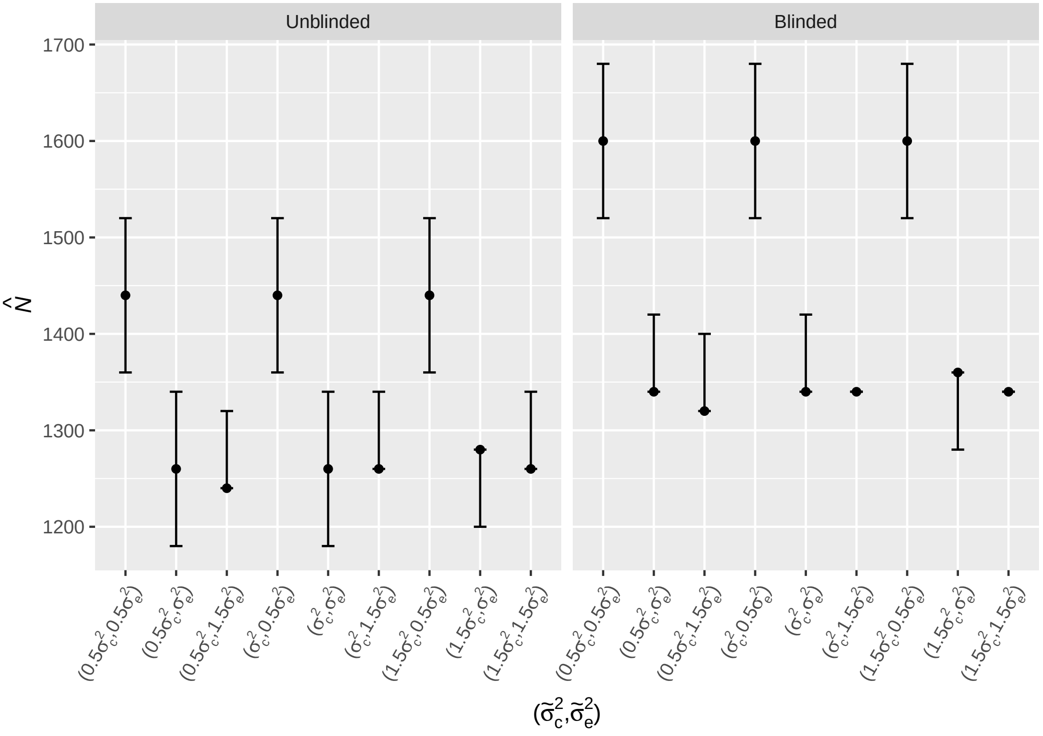

(b) $\tau = \delta$

Supplementary Figure 6: Distributions of $\hat{N}$ are shown via their 25th, 50th and 75th percentiles across the replicate simulations. Results are presented for scenarios with $\tau = 0$ and $\tau = \delta$, using the blinded ($\tau_* = 0$) and unblinded re-estimation procedures. They are given for Trial Design Setting 2 ($t = 5$), for a selection of possible values for the assumed variance parameters, with $n_{\text{min}} = 1$ and $n_{\text{max}} = 1000$.

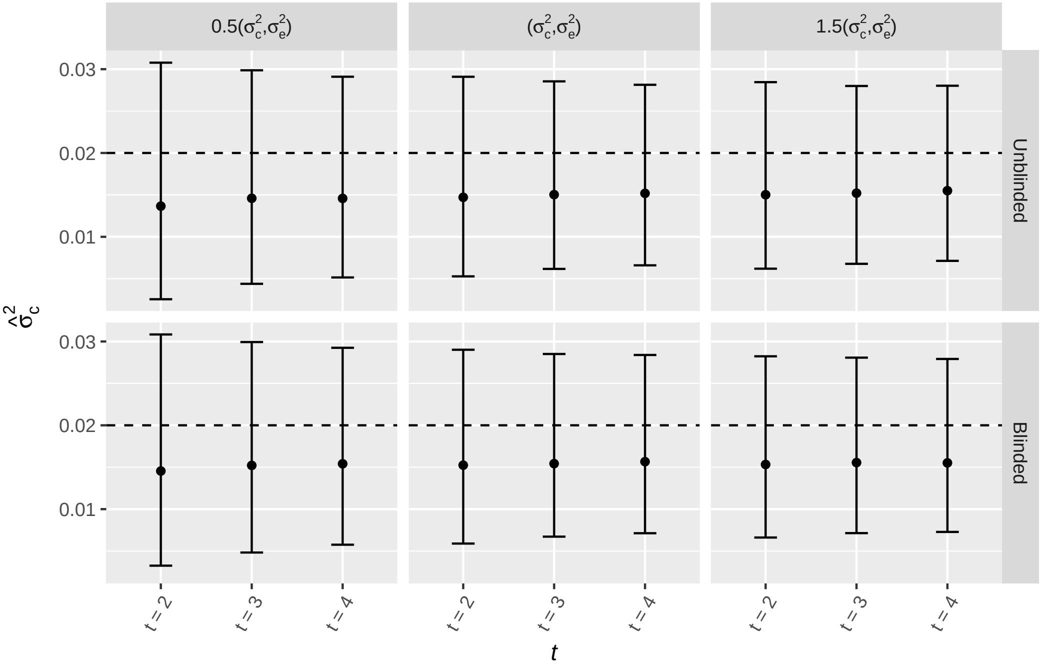

(a) $\hat{\sigma}_c^2$

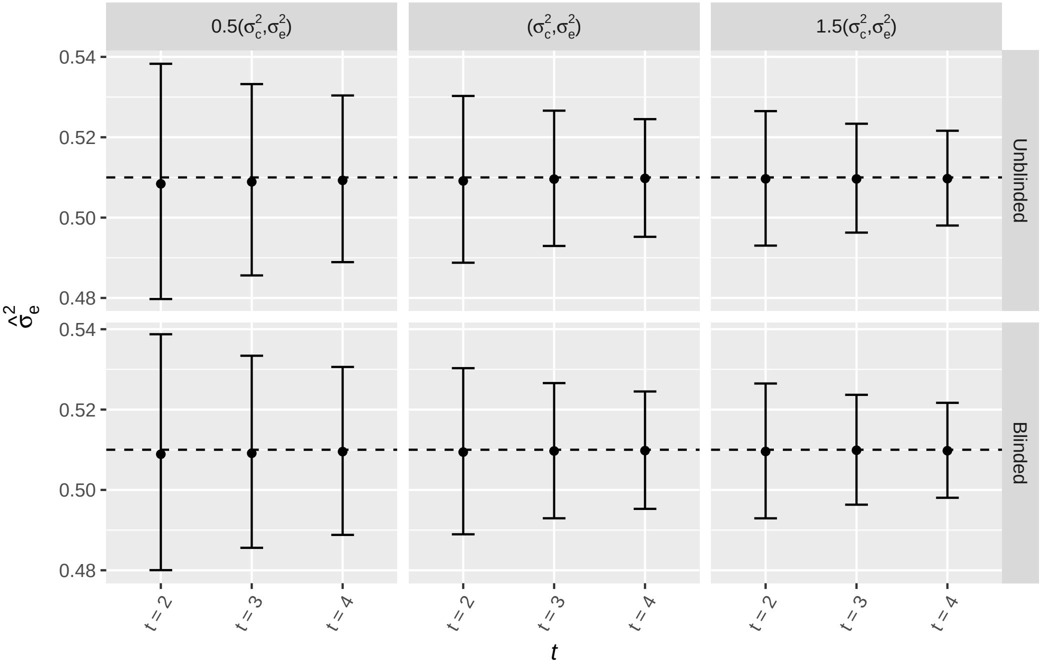

(b) $\hat{\sigma}_e^2$

Supplementary Figure 7: Distributions of $\hat{\sigma}_c^2$ and $\hat{\sigma}_e^2$ are shown via their 25th, 50th and 75th percentiles across the replicate simulations. Results are presented for scenarios with $\tau = 0$, using the blinded ($\tau_* = 0$) and unblinded re-estimation procedures. They are given for Trial Design Setting 1, for a selection of possible values for the assumed variance parameters and values for t, with $n_{\text{min}} = 1$ and $n_{\text{max}} = 1000$.

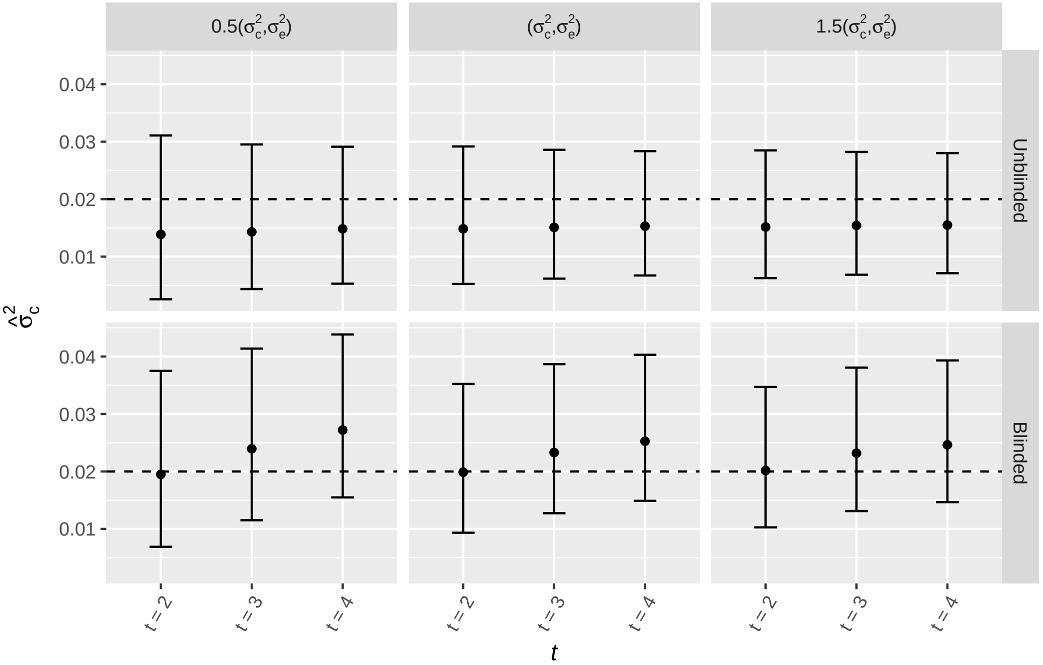

(a) $\hat{\sigma}_c^2$

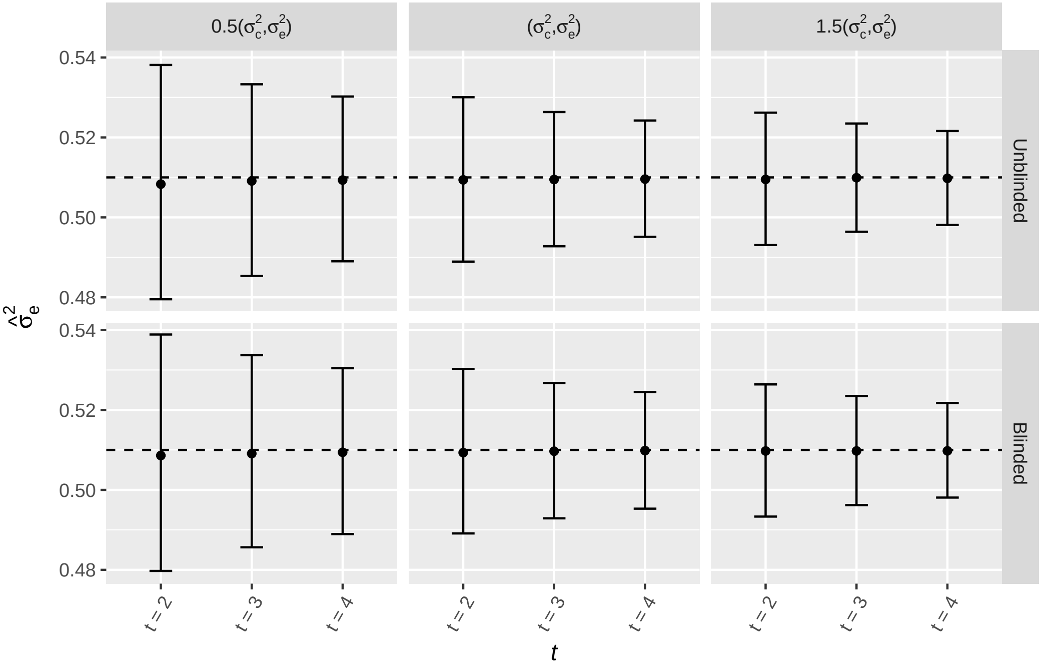

(b) $\hat{\sigma}_e^2$

Supplementary Figure 8: Distributions of $\hat{\sigma}_c^2$ and $\hat{\sigma}_e^2$ are shown via their 25th, 50th and 75th percentiles across the replicate simulations. Results are presented for scenarios with $\tau = \delta$, using the blinded ($\tau_* = 0$) and unblinded re-estimation procedures. They are given for Trial Design Setting 1 ($t = 3$), for a selection of possible values for the assumed variance parameters and values for t, with $n_{\text{min}} = 1$ and $n_{\text{max}} = 1000$.

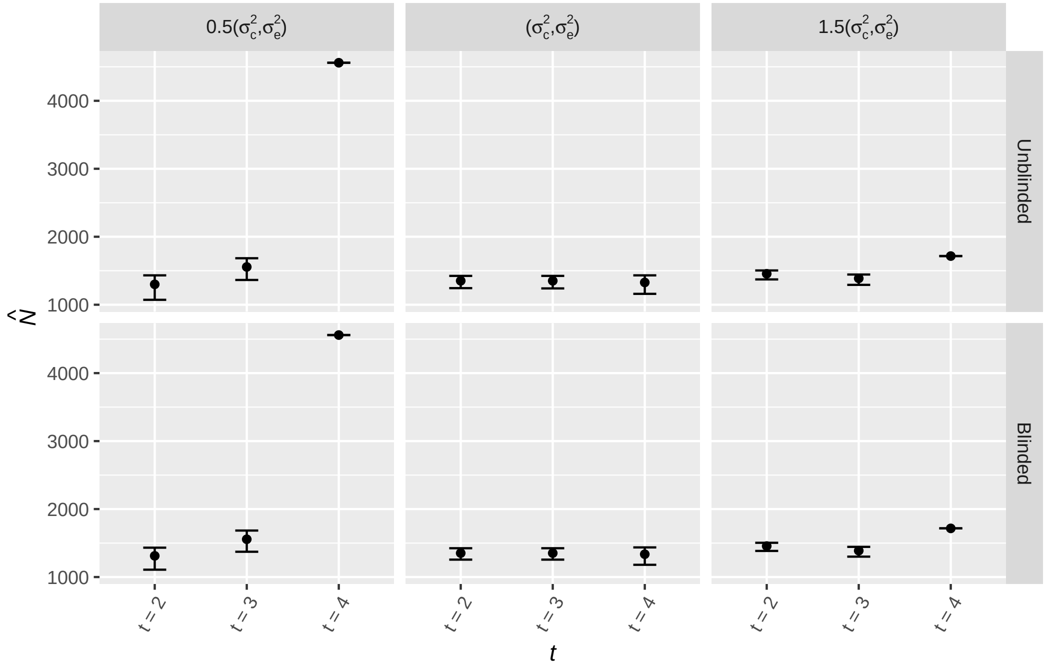

(a) $\tau = 0$

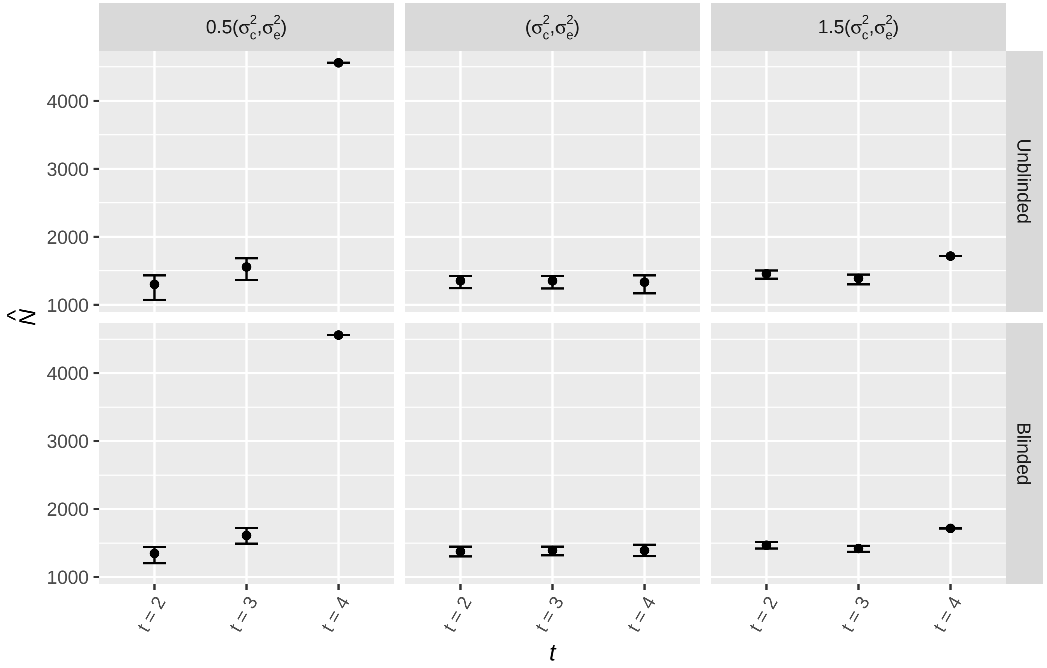

(b) $\tau = \delta$

Supplementary Figure 9: Distributions of $\hat{N}$ are shown via their 25th, 50th and 75th percentiles across the replicate simulations. Results are presented for scenarios with $\tau = 0$ and $\tau = \delta$, using the blinded ($\tau_* = 0$) and unblinded re-estimation procedures. They are given for Trial Design Setting 1 ($t = 3$), for a selection of possible values for the assumed variance parameters and values for t, with $n_{\text{min}} = 1$ and $n_{\text{max}} = 1000$.

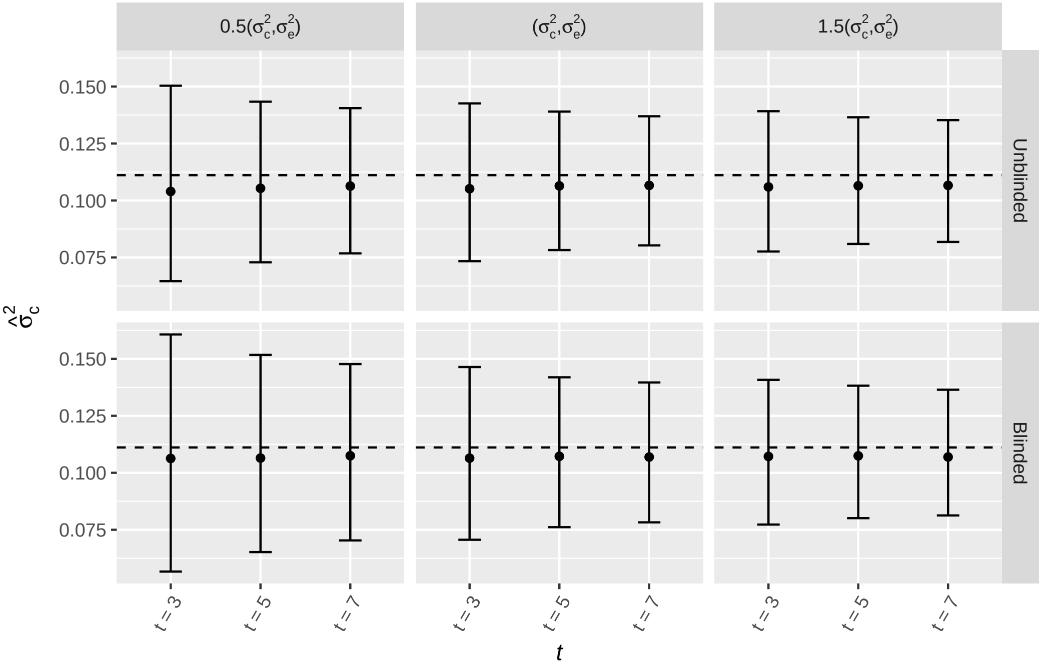

(a) $\hat{\sigma}_c^2$

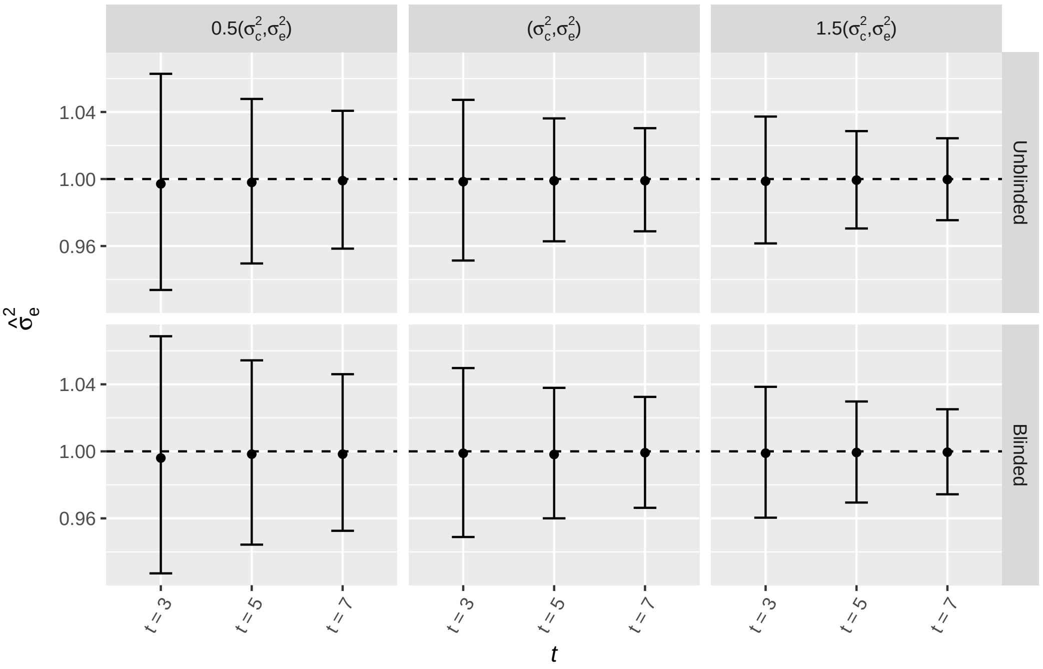

(b) $\hat{\sigma}_e^2$

Supplementary Figure 10: Distributions of $\hat{\sigma}_c^2$ and $\hat{\sigma}_e^2$ are shown via their 25th, 50th and 75th percentiles across the replicate simulations. Results are presented for scenarios with $\tau = 0$, using the blinded ($\tau_* = 0$) and unblinded re-estimation procedures. They are given for Trial Design Setting 2 ($t = 5$), for a selection of possible values for the assumed variance parameters and values for t, with $n_{\text{min}} = 1$ and $n_{\text{max}} = 1000$.

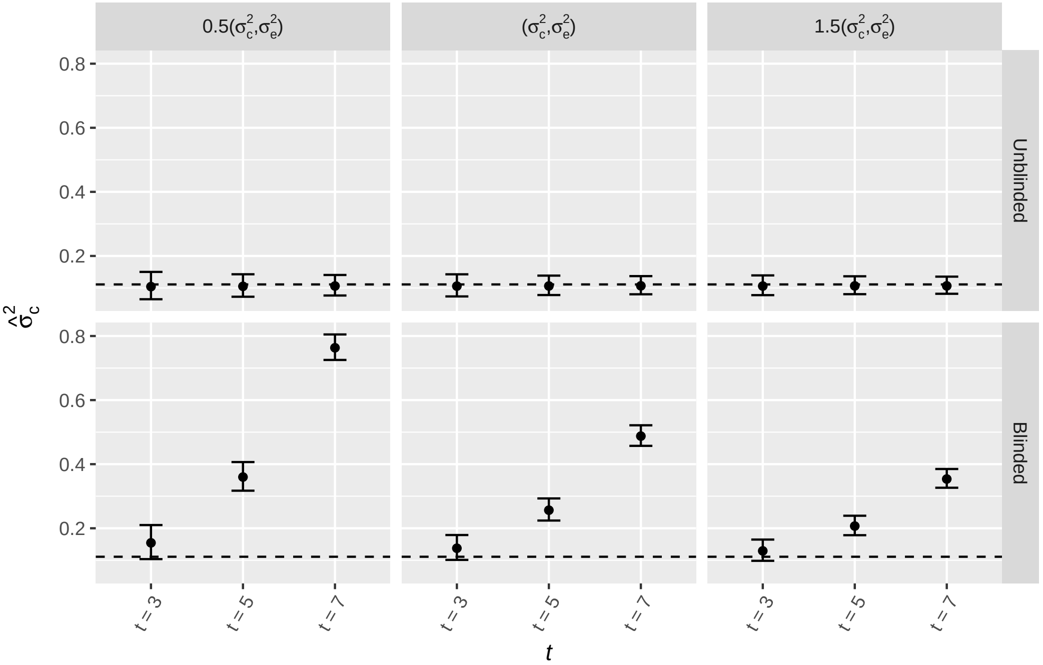

(a) $\hat{\sigma}_c^2$

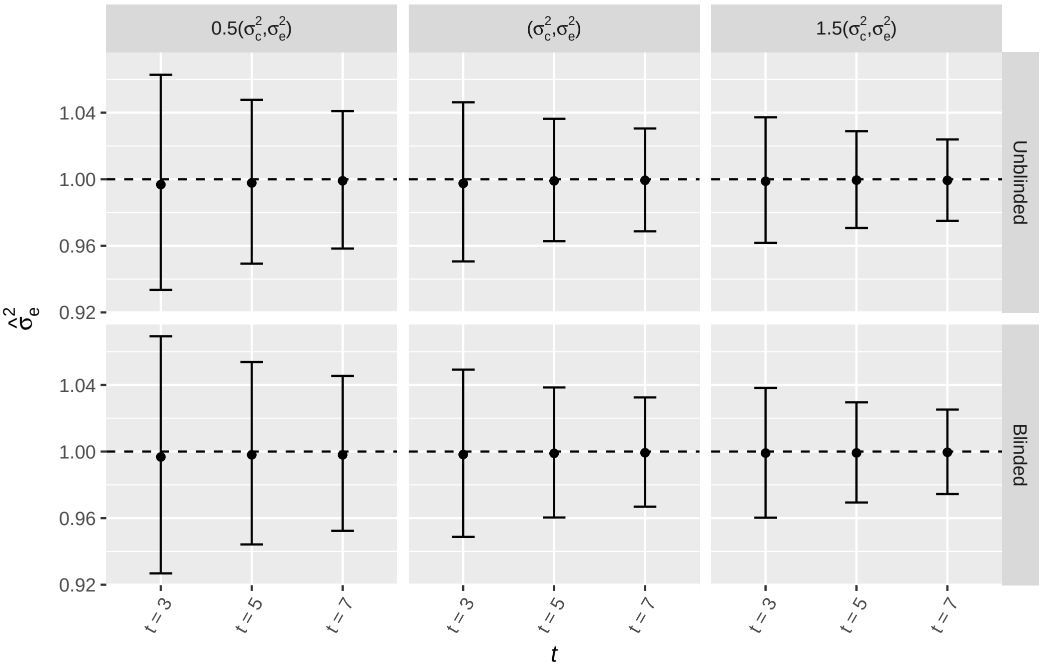

(b) $\hat{\sigma}_e^2$

Supplementary Figure 11: Distributions of $\hat{\sigma}_c^2$ and $\hat{\sigma}_e^2$ are shown via their 25th, 50th and 75th percentiles across the replicate simulations. Results are presented for scenarios with $\tau = \delta$, using the blinded ($\tau_* = 0$) and unblinded re-estimation procedures. They are given for Trial Design Setting 2 ($t = 5$), for a selection of possible values for the assumed variance parameters and values for t, with $n_{\text{min}} = 1$ and $n_{\text{max}} = 1000$.

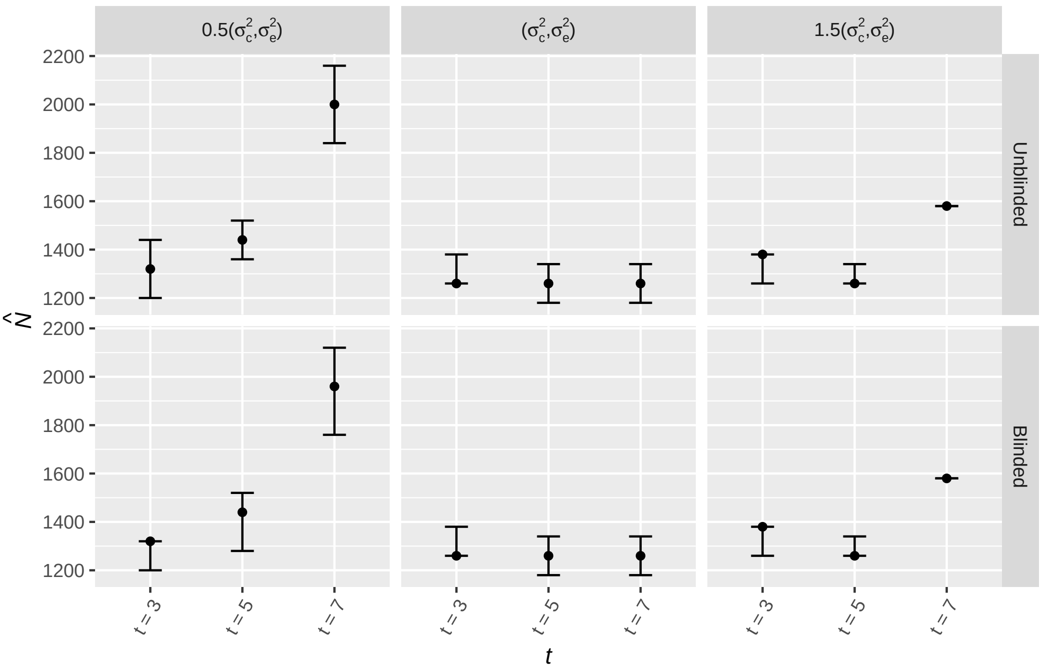

(a) $\tau = 0$

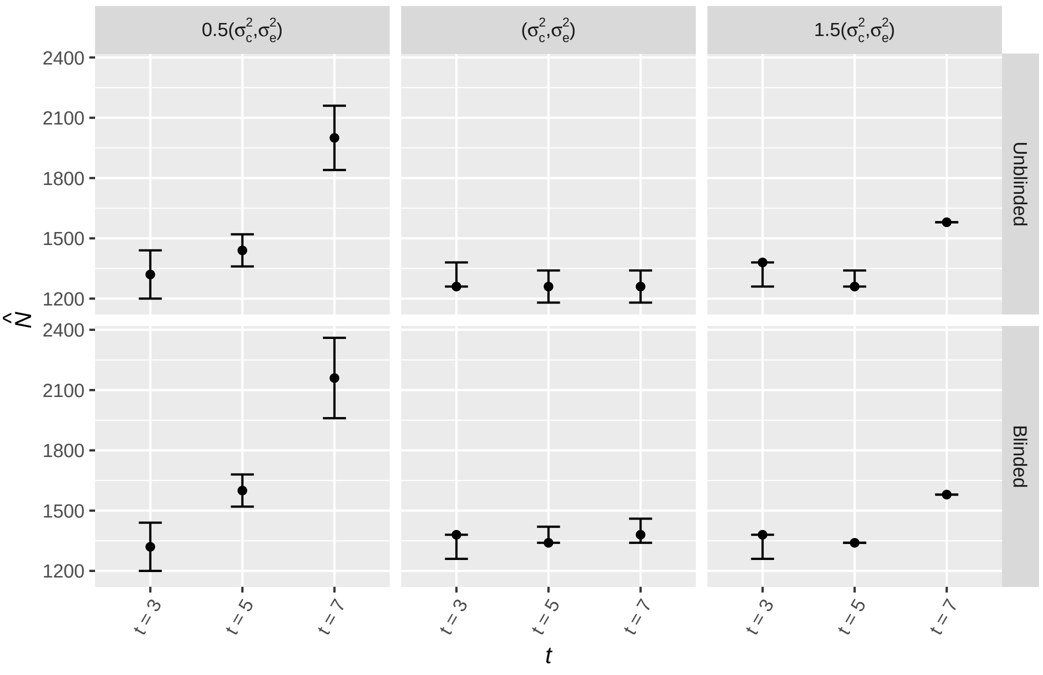

(b) $\tau = \delta$

Supplementary Figure 12: Distributions of $\hat{N}$ are shown via their 25th, 50th and 75th percentiles across the replicate simulations. Results are presented for scenarios with $\tau = 0$ and $\tau = \delta$, using the blinded ($\tau_* = 0$) and unblinded re-estimation procedures. They are given for Trial Design Setting 2 ($t = 5$), for a selection of possible values for the assumed variance parameters and values for t, with $n_{\text{min}} = 1$ and $n_{\text{max}} = 1000$.

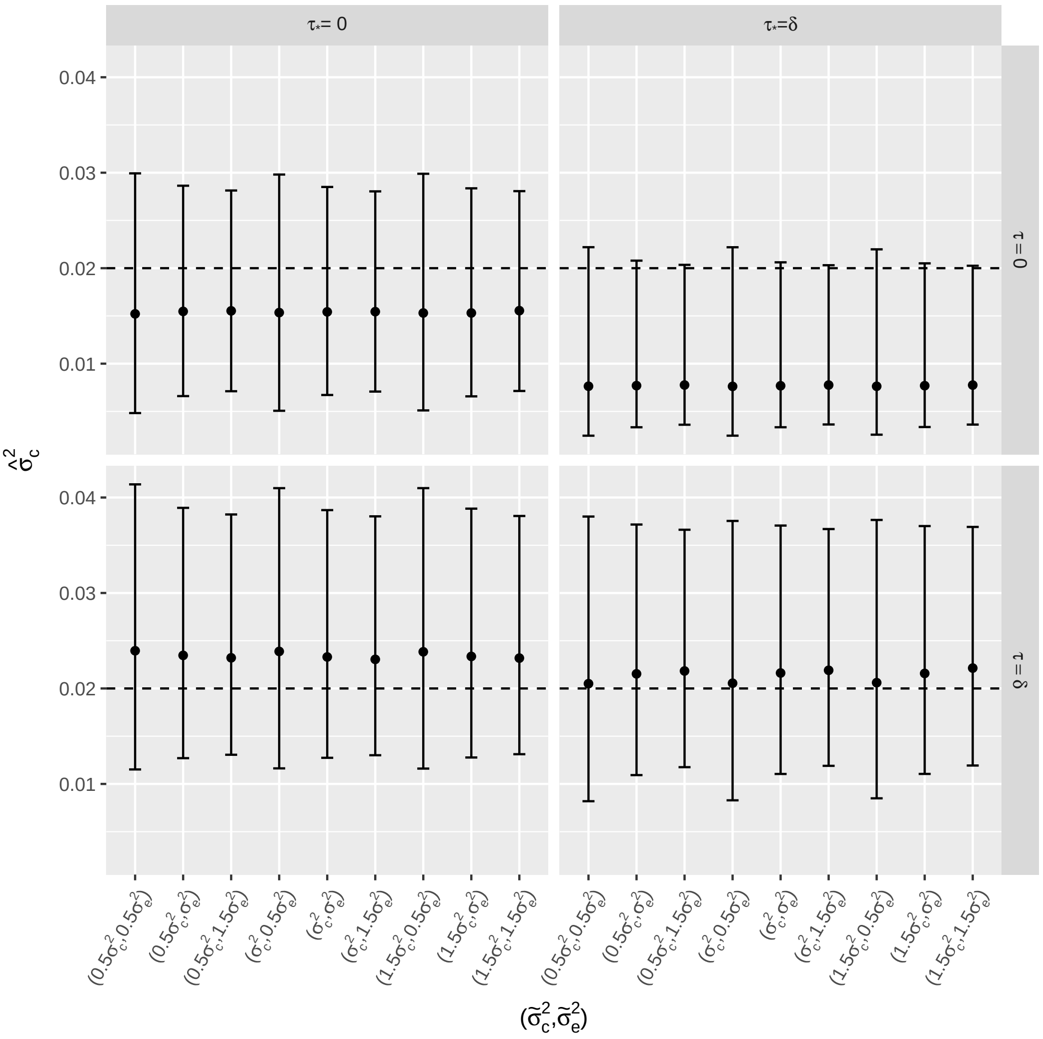

Supplementary Figure 13: Distributions of $\hat{\sigma_c}^2$ are shown via their 25th, 50th and 75th percentiles across the replicate simulations. Results are presented for scenarios with $\tau = 0$ and $\tau = \delta$, using the blinded ($\tau_* = 0$ and $\tau_* = \delta$) re-estimation procedure. They are given for Trial Design Setting 1 ($t = 3$), for a selection of possible values for the assumed variance parameters, with $n_{\text{min}} = 1$ and $n_{\text{max}} = 1000$.

and Supplementary Table 2, which displays the median required sample sizes. Moreover, the distributions of $\hat{N}$ are given in Supplementary Figures 17-20.

As would be expected, we observe that when n is only allowed to increase from its initial value ($n_{\text{min}} = n_{\text{init}},\ n_{\text{max}} = 1000$), the power of our procedures improves beyond that given in Table 1. Of course though, examining Supplementary Table 2, this is at a cost of increased required sample sizes.

Similarly, when $n_{\text{min}} = 1,\ n_{\text{max}} = n_{\text{init}}$, the EP of the procedures remains high when the variance parameters are over-specified, with the median value of $\hat{N}$ reducing to a preferable level. However, when the variance parameters are under-specified, allowing the value of n to only decrease following the interim analysis leads to a substantial loss of power in certain circumstances, particular in TDS1.

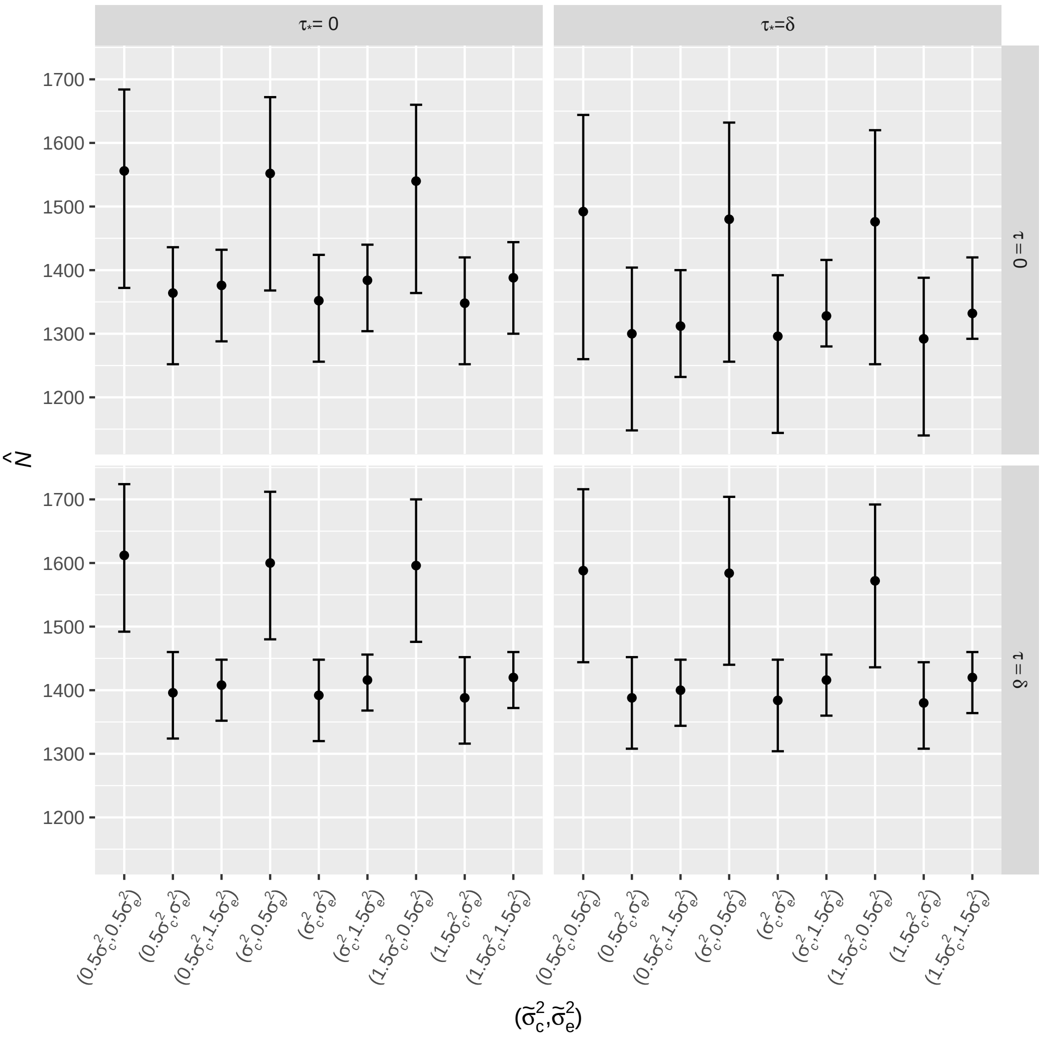

Supplementary Figure 14: Distributions of $\hat{N}$ are shown via their 25th, 50th and 75th percentiles across the replicate simulations. Results are presented for scenarios with $\tau = 0$ and $\tau = \delta$, using the blinded ($\tau_* = 0$ and $\tau_* = \delta$) re-estimation procedure. They are given for Trial Design Setting 1 ($t = 3$), for a selection of possible values for the assumed variance parameters, with $n_{\text{min}} = 1$ and $n_{\text{max}} = 1000$.

S.M.8 Performance assuming $\sigma_c^2 = 0$

Given that, in general, we would expect our blinded estimate of σ_c^2 to be biased due to $\tau_* \neq \tau$, it is interesting to ask how well a blinded procedure that simply assumes $\sigma_c^2 = \hat{\sigma}_c^2 = 0$ in the re-estimation procedure could perform. In this section it is this we examine.

For TDS1 ($t = 3$) and TDS2 ($t = 5$), with $\tilde{\sigma}_e^2 \in \{0.5\sigma_e^2, \sigma_e^2, 1.5\sigma_e^2\}$, $\tau \in \{0, \delta\}$, $n_{\text{min}} = 1$, $n_{\text{max}} = 1000$, and taking $\tau_* = 0$, we examine the performance of the blinded re-estimation procedure when we set $\tilde{\sigma}_c^2 = \hat{\sigma}_c^2 = 0$. We contrast our findings to the results from Section 3.1, and specifically Table 1. Our findings are presented in Supplementary Table 3.

We can see that assuming $\sigma_c^2 = 0$ leads to a large drop in power relative to even assuming $\tilde{\sigma}_c^2 = 0.5\sigma_c^2$. Consequently, we would caution against assuming that between cluster variance to be zero, even what it is anticipated

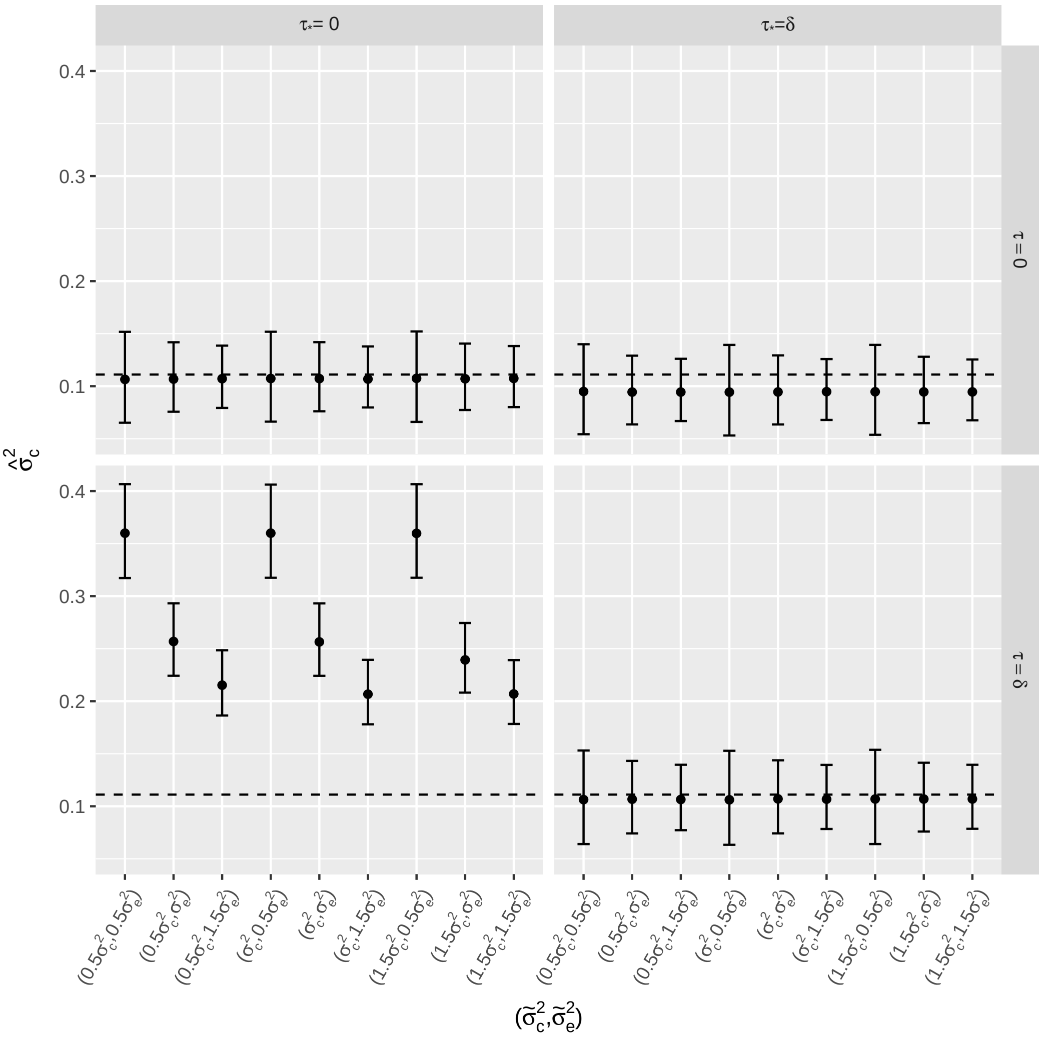

Supplementary Figure 15: Distributions of $\hat{\sigma_c}^2$ are shown via their 25th, 50th and 75th percentiles across the replicate simulations. Results are presented for scenarios with $\tau = 0$ and $\tau = \delta$, using the blinded ($\tau_* = 0$ and $\tau_* = \delta$) re-estimation procedure. They are given for Trial Design Setting 2 ($t = 5$), for a selection of possible values for the assumed variance parameters, with $n_{\text{min}} = 1$ and $n_{\text{max}} = 1000$.

to be small.

S.M.9 Performance when $\sigma_c^2 = \sigma_e^2$

As can be seen in Table 1 and Supplementary Table 1, changing the value of $\tilde{\sigma}_c^2$ (or even $\tilde{\sigma}_e^2$ in the case of Table 1) has only a very minor effect on the performance of the re-estimation procedures when all other variables are held constant. This could be a consequence of the fact that $\sigma_c^2 \ll \sigma_e^2$. In practice, small values of the ICC would be common. However, it is informative to ask how well the re-estimation procedures might perform for an extremely large value of ρ. Therefore, in this section we consider the performance of the re-estimation procedures in cases where $\sigma_c^2 = \sigma_e^2 = \sigma^2$, say.

Precisely, we consider scenarios in which $\sigma^2 \in \{0.25, 0.5, 1, 2.5, 5, 10\}$, $(\tilde{\sigma}_c^2, \tilde{\sigma}_e^2) = (\tilde{\sigma}^2, \tilde{\sigma}^2) \in$

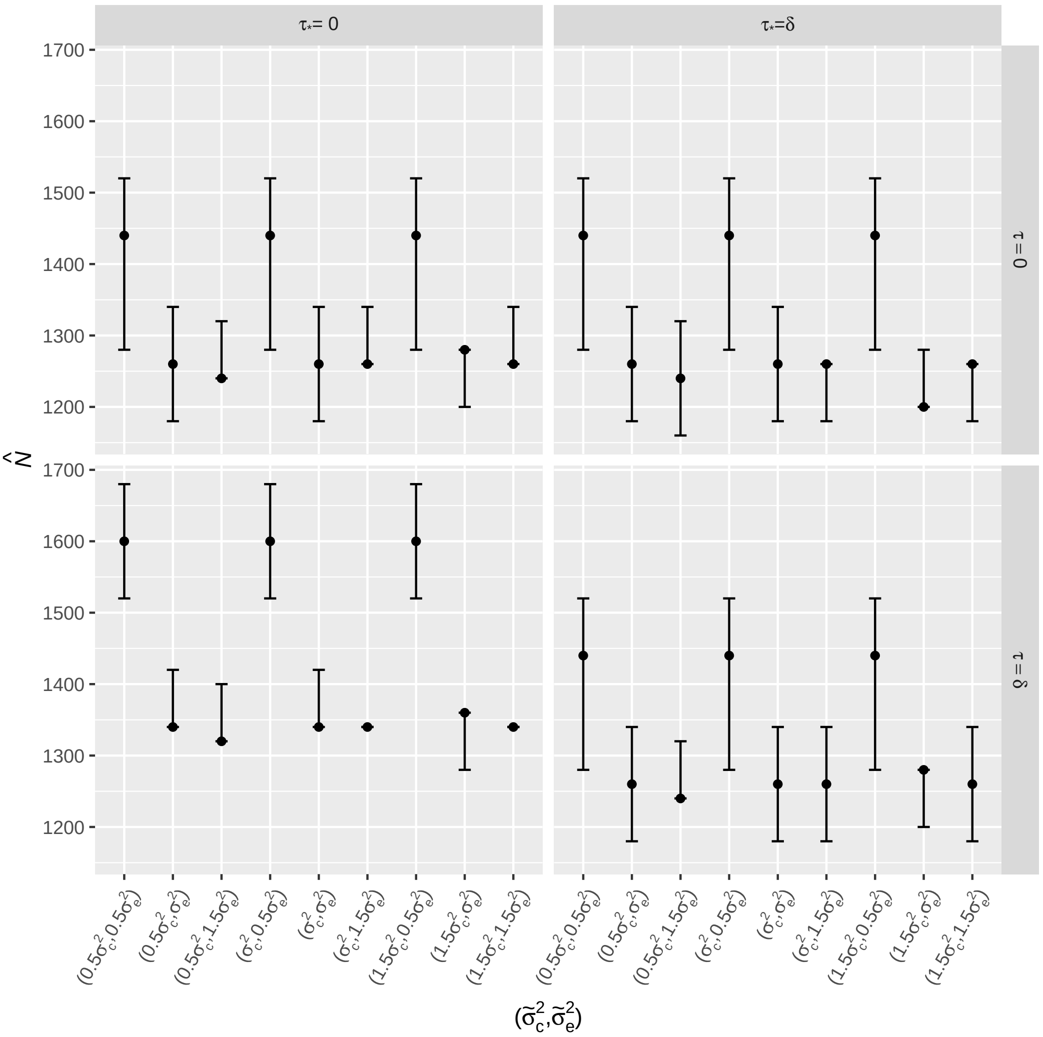

Supplementary Figure 16: Distributions of $\hat{N}$ are shown via their 25th, 50th and 75th percentiles across the replicate simulations. Results are presented for scenarios with $\tau = 0$ and $\tau = \delta$, using the blinded ($\tau_* = 0$ and $\tau_* = \delta$) re-estimation procedure. They are given for Trial Design Setting 2 ($t = 5$), for a selection of possible values for the assumed variance parameters, with $n_{\text{min}} = 1$ and $n_{\text{max}} = 1000$.

$\{0.5(\sigma^2, \sigma^2), (\sigma^2, \sigma^2), 1.5(\sigma^2, \sigma^2)\}$, and $\tau \in \{0, \delta\}$, with $n_{\text{min}} = 1$ and $n_{\text{max}} = 1000$. Performance of both the blinded ($\tau_* = 0$) and unblinded procedures is considered, with $n_{\text{min}} = 1$ and $n_{\text{max}} = 1000$, and with the values of $\boldsymbol{X}$, α and β set as in Section 2.3 for TDS1 ($t = 3$) and TDS2 ($t = 5$) respectively.

Moreover, δ is here taken to be different from the 0.2 and 0.247 values used for TDS1 and TDS2 in the rest of the article. It is chosen in each simulation scenario for TDS1 to be the value that implies a per cluster per period sample size of $n = 50$ would actually be required in a fixed sample trial for the desired operating characteristics. Similarly, for TDS2 it is chosen such that $n = 10$ would be required. This ensures that the effect on performance as σ^2 increases can be more readily assessed without the need to account for the underlying requisite sample size. Our results are provided in Supplementary Table 4.

It is clear from Supplementary Table 4 that the values of $\tilde{\sigma}^2$ and σ^2 have little effect on the resultant power. Thus, the SSRE procedures do still perform well even in the case of a large ICC. It is important to note however

Supplementary Table 1: Empirical type-I error rates ($\tau = 0$) and power ($\tau = \delta$) of the blinded ($\tau_* = 0$) and unblinded re-estimation procedures are shown. Results are given for Trial Design Settings 1 ($t = 3$) and 2 ($t = 5$), for a selection of possible values for the assumed variance parameters, and two different possible combinations of n_{min} and n_{max}.

		$n_{\text{min}} = n_{\text{init}},\ n_{\text{max}} = 1000$				$n_{\text{min}} = 1,\ n_{\text{max}} = n_{\text{init}}$			
		$\tau = 0$		$\tau = \delta$		$\tau = 0$		$\tau = \delta$	
$\tilde{\sigma}_c^2$	$\tilde{\sigma}_e^2$	Bl.	Unbl.	Bl.	Unbl.	Bl.	Unbl.	Bl.	Unbl.
Trial Design Setting 1									
$0.5\sigma_c^2$	$0.5\sigma_e^2$	0.0576	0.0595	0.8844	0.8777	0.0583	0.0604	0.6913	0.8766
$0.5\sigma_c^2$	σ_e^2	0.0586	0.0585	0.9017	0.9005	0.0593	0.0657	0.8764	0.8832
$0.5\sigma_c^2$	$1.5\sigma_e^2$	0.0574	0.0562	0.9689	0.9699	0.0624	0.0662	0.8930	0.8938
σ_c^2	$0.5\sigma_e^2$	0.0569	0.0608	0.8843	0.8779	0.0577	0.0598	0.7031	0.8779
σ_c^2	σ_e^2	0.0579	0.0585	0.9053	0.9048	0.0599	0.0652	0.8830	0.8863
σ_c^2	$1.5\sigma_e^2$	0.0574	0.0563	0.9734	0.9751	0.0650	0.0647	0.8946	0.8952
$1.5\sigma_c^2$	$0.5\sigma_e^2$	0.0574	0.0611	0.8821	0.8780	0.0587	0.0602	0.7127	0.8782
$1.5\sigma_c^2$	σ_e^2	0.0590	0.0591	0.9086	0.9091	0.0596	0.0644	0.8830	0.8848
$1.5\sigma_c^2$	$1.5\sigma_e^2$	0.0554	0.0568	0.9737	0.9745	0.0642	0.0647	0.8978	0.8971
Trial Design Setting 2									
$0.5\sigma_c^2$	$0.5\sigma_e^2$	0.0257	0.0266	0.8263	0.8007	0.0268	0.0265	0.8262	0.8012
$0.5\sigma_c^2$	σ_e^2	0.0266	0.0265	0.8280	0.8138	0.0267	0.0273	0.8264	0.8071
$0.5\sigma_c^2$	$1.5\sigma_e^2$	0.0264	0.0253	0.9123	0.9121	0.0267	0.0260	0.8283	0.8091
σ_c^2	$0.5\sigma_e^2$	0.0254	0.0269	0.8285	0.8024	0.0260	0.0271	0.8276	0.8008
σ_c^2	σ_e^2	0.0264	0.0266	0.8261	0.8141	0.0261	0.0268	0.8272	0.8059
σ_c^2	$1.5\sigma_e^2$	0.0259	0.0257	0.9340	0.9338	0.0270	0.0265	0.8306	0.8123
$1.5\sigma_c^2$	$0.5\sigma_e^2$	0.0265	0.0260	0.8274	0.8000	0.0266	0.0255	0.8288	0.8011
$1.5\sigma_c^2$	σ_e^2	0.0260	0.0248	0.8491	0.8470	0.0270	0.0272	0.8270	0.8066
$1.5\sigma_c^2$	$1.5\sigma_e^2$	0.0261	0.0262	0.9347	0.9340	0.0255	0.0271	0.8276	0.8125

that we consider here only the case where $n_{\text{min}} = 1$ and $n_{\text{max}} = 1000$. It could well be that the designs ETI and EP perform particularly badly for large ICCs if the value of n_{final} is not allowed to increase and decrease.

References

Hussey, M.A. and Hughes, J.P. (2007). Design and analysis of stepped wedge cluster randomised trials. *Contemporary Clinical Trials* **28**, 181–191.

Supplementary Table 2: Median values of the final total required sample size ($\hat{N}$) are shown for $\tau = 0$ and $\tau = \delta$ using the blinded ($\tau_* = 0$) and unblinded re-estimation procedures. Results are given for Trial Design Settings 1 ($t = 3$) and 2 ($t = 5$), for a selection of possible values for the assumed variance parameters, and two different possible combinations of n_{min} and n_{max}.

		$n_{\text{min}} = n_{\text{init}},\ n_{\text{max}} = 1000$				$n_{\text{min}} = 1,\ n_{\text{max}} = n_{\text{init}}$			
		$\tau = 0$		$\tau = \delta$		$\tau = 0$		$\tau = \delta$	
$\tilde{\sigma}_c^2$	$\tilde{\sigma}_e^2$	Bl.	Unbl.	Bl.	Unbl.	Bl.	Unbl.	Bl.	Unbl.
Trial Design Setting 1									
$0.5\sigma_c^2$	$0.5\sigma_e^2$	1556	1556	1612	1556	1556	1548	700	1548
$0.5\sigma_c^2$	σ_e^2	1356	1356	1396	1356	1364	1356	1340	1356
$0.5\sigma_c^2$	$1.5\sigma_e^2$	2040	2040	2040	2040	1376	1376	1408	1376
σ_c^2	$0.5\sigma_e^2$	1552	1544	1600	1544	1544	1544	720	1544
σ_c^2	σ_e^2	1400	1400	1400	1400	1352	1352	1392	1352
σ_c^2	$1.5\sigma_e^2$	2120	2120	2120	2120	1384	1384	1416	1384
$1.5\sigma_c^2$	$0.5\sigma_e^2$	1540	1532	1588	1532	1540	1532	740	1532
$1.5\sigma_c^2$	σ_e^2	1420	1420	1420	1420	1348	1348	1388	1348
$1.5\sigma_c^2$	$1.5\sigma_e^2$	2140	2140	2140	2140	1388	1388	1420	1388
Trial Design Setting 2									
$0.5\sigma_c^2$	$0.5\sigma_e^2$	1440	1440	1600	1440	1440	1440	1600	1440
$0.5\sigma_c^2$	σ_e^2	1260	1260	1340	1260	1260	1260	1340	1260
$0.5\sigma_c^2$	$1.5\sigma_e^2$	1800	1800	1800	1800	1240	1240	1320	1240
σ_c^2	$0.5\sigma_e^2$	1440	1440	1600	1440	1440	1440	1600	1440
σ_c^2	σ_e^2	1260	1260	1340	1260	1260	1260	1340	1260
σ_c^2	$1.5\sigma_e^2$	1980	1980	1980	1980	1260	1260	1340	1260
$1.5\sigma_c^2$	$0.5\sigma_e^2$	1440	1440	1600	1440	1440	1440	1600	1440
$1.5\sigma_c^2$	σ_e^2	1440	1440	1440	1440	1280	1280	1360	1280
$1.5\sigma_c^2$	$1.5\sigma_e^2$	1980	1980	1980	1980	1260	1260	1340	1260

Supplementary Table 3: Empirical type-I error rates ($\tau = 0$) and power ($\tau = \delta$) of the blinded re-estimation procedure are shown. Results are given for Trial Design Settings 1 ($t = 3$) and 2 ($t = 5$), for a selection of possible values for the assumed residual variance, when $n_{\text{min}} = 1$ and $n_{\text{max}} = 1000$. In all scenarios here, we assume that $\tilde{\sigma}_c^2 = \hat{\sigma}_c^2 = 0$.

$\tilde{\sigma}_e^2$	$\tau = 0$	$\tau = \delta$
Trial Design Setting 1		
$0.5\sigma_e^2$	0.0605	0.7750
σ_e^2	0.0611	0.7806
$1.5\sigma_e^2$	0.0686	0.7386
Trial Design Setting 2		
$0.5\sigma_e^2$	0.0266	0.6610
σ_e^2	0.0266	0.6719
$1.5\sigma_e^2$	0.0271	0.6494

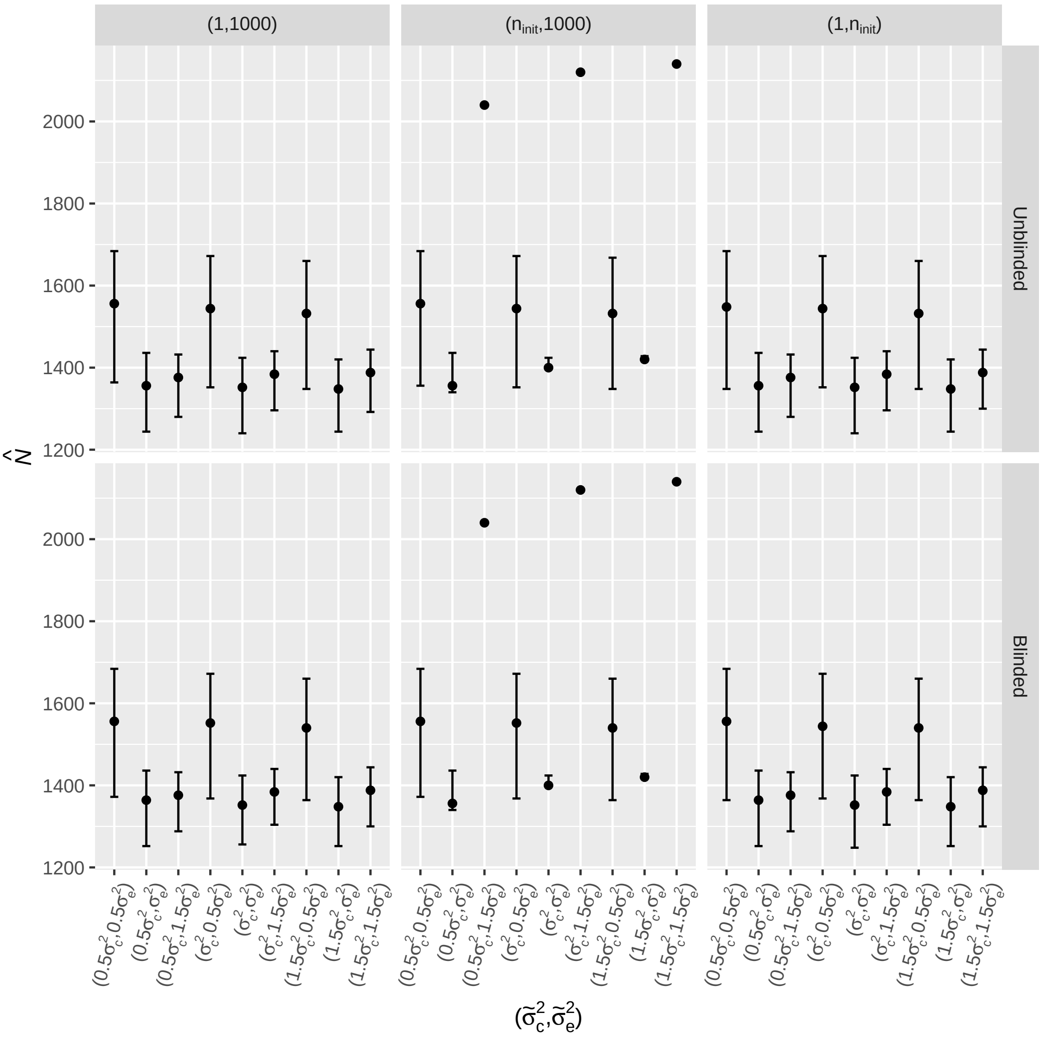

Supplementary Figure 17: Distributions of $\hat{N}$ are shown via their 25th, 50th and 75th percentiles across the replicate simulations. Results are presented for scenarios with $\tau = 0$, using the blinded ($\tau_* = 0$) and unblinded re-estimation procedures. They are given for Trial Design Setting 1 ($t = 3$), for a selection of possible values for the assumed variance parameters, and a selection of possible values for n_{min} and n_{max}.

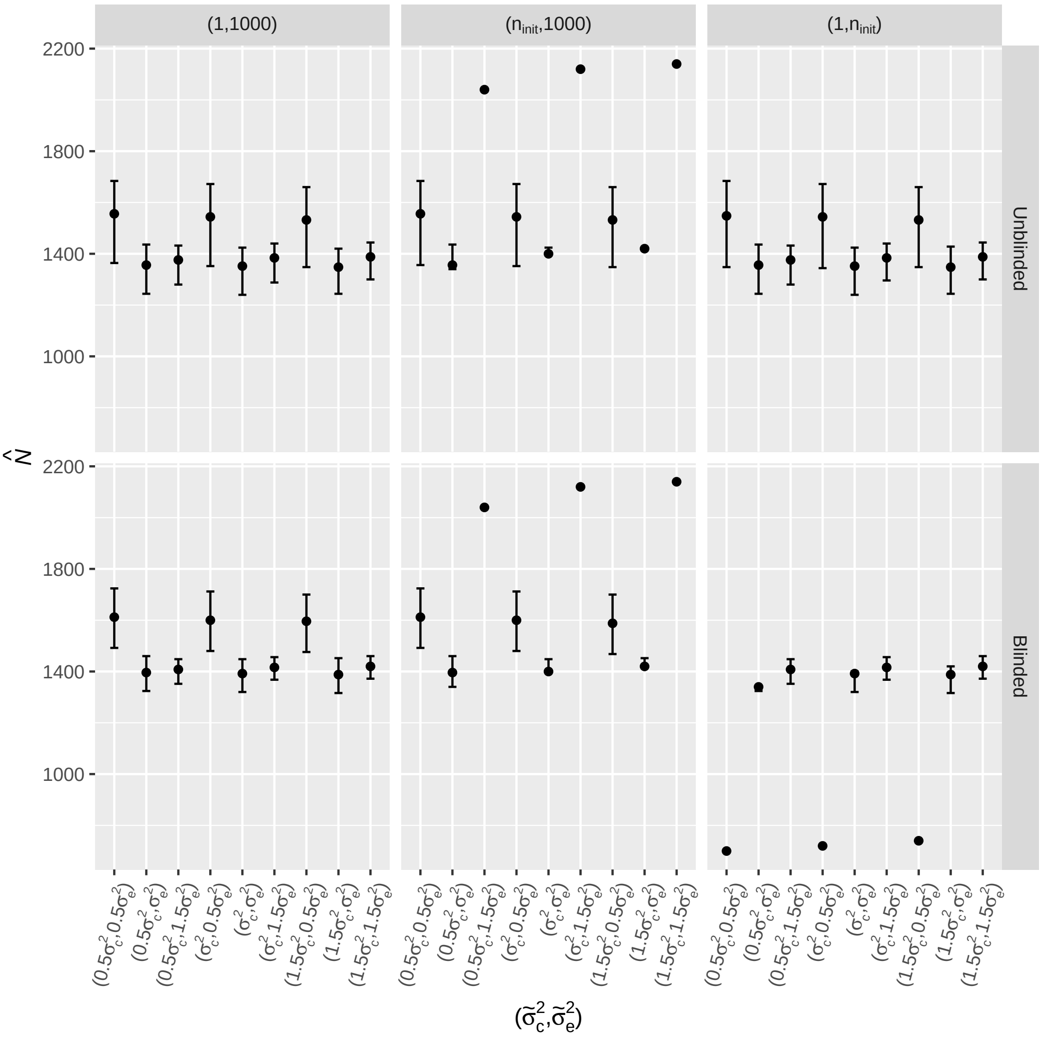

Supplementary Figure 18: Distributions of $\hat{N}$ are shown via their 25th, 50th and 75th percentiles across the replicate simulations. Results are presented for scenarios with $\tau = \delta$, using the blinded ($\tau_* = 0$) and unblinded re-estimation procedures. They are given for Trial Design Setting 1 ($t = 3$), for a selection of possible values for the assumed variance parameters, and a selection of possible values for n_{min} and n_{max}.

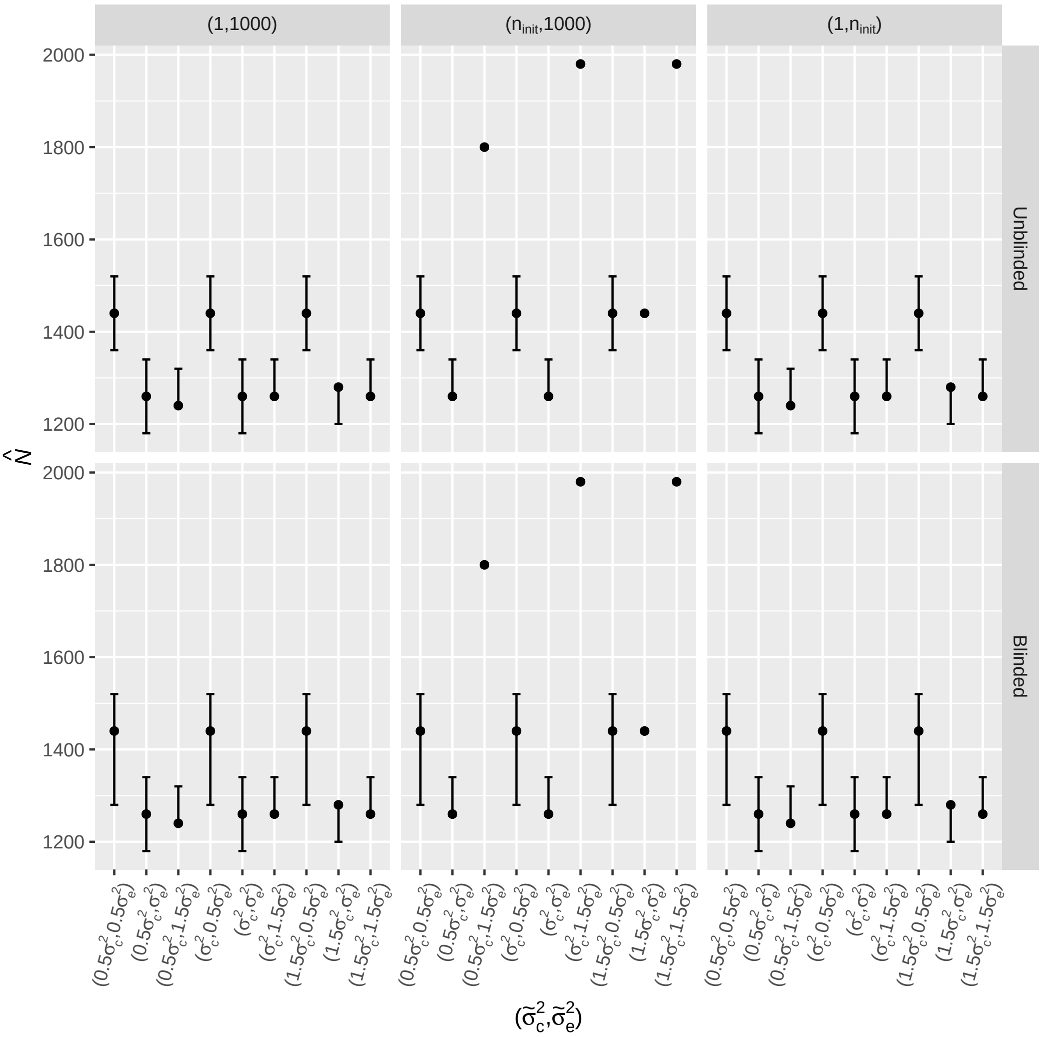

Supplementary Figure 19: Distributions of $\hat{N}$ are shown via their 25th, 50th and 75th percentiles across the replicate simulations. Results are presented for scenarios with $\tau = 0$, using the blinded ($\tau_* = 0$) and unblinded re-estimation procedures. They are given for Trial Design Setting 2 ($t = 5$), for a selection of possible values for the assumed variance parameters, and a selection of possible values for n_{min} and n_{max}.

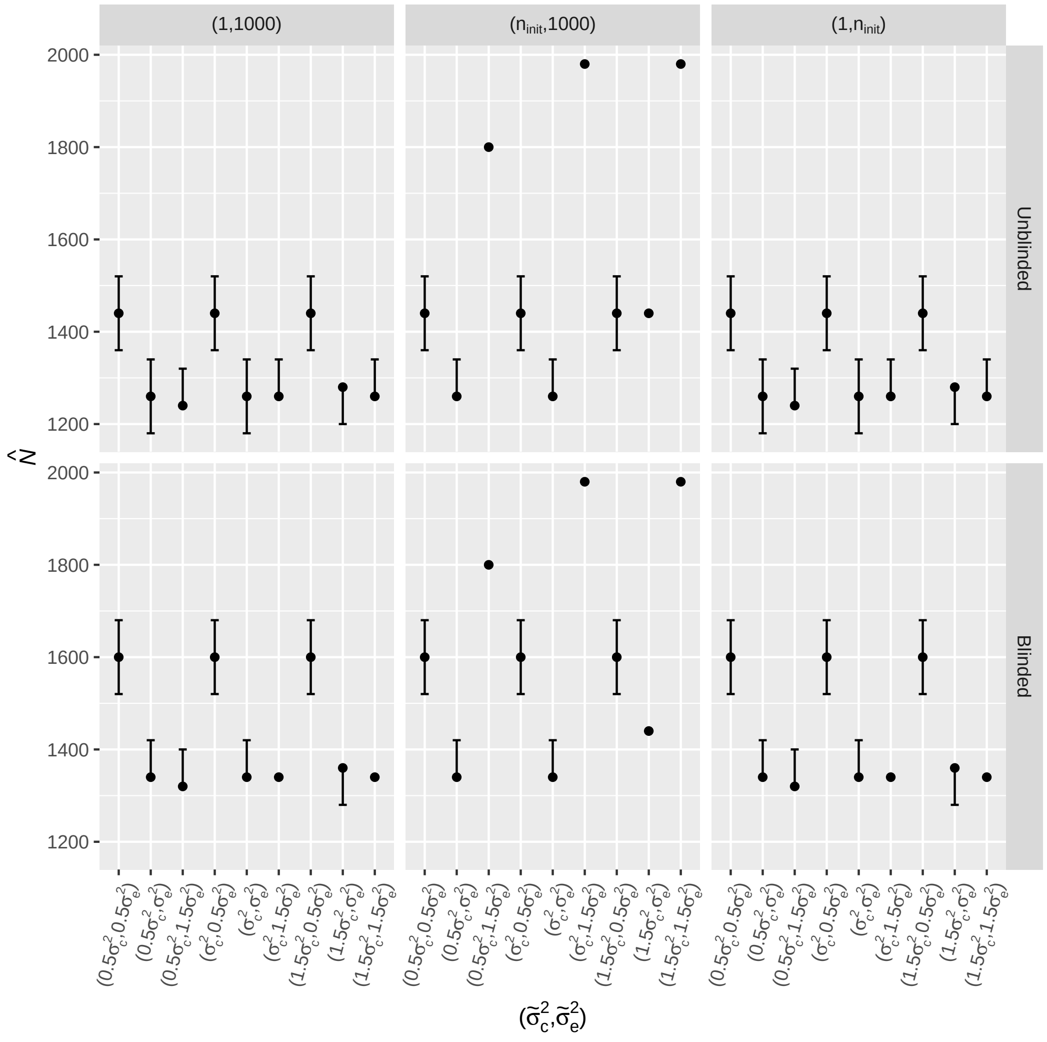

Supplementary Figure 20: Distributions of $\hat{N}$ are shown via their 25th, 50th and 75th percentiles across the replicate simulations. Results are presented for scenarios with $\tau = \delta$, using the blinded ($\tau_* = 0$) and unblinded re-estimation procedures. They are given for Trial Design Setting 2 ($t = 5$), for a selection of possible values for the assumed variance parameters, and a selection of possible values for n_{min} and n_{max}.

Supplementary Table 4: Empirical type-I error rates ($\tau = 0$) and power ($\tau = \delta$) of the blinded ($\tau_* = 0$) and unblinded re-estimation procedures are shown. Results are given for Trial Design Settings 1 ($t = 3$) and 2 ($t = 5$), for scenarios with $\sigma_c^2 = \sigma_e^2 = \sigma^2$ and $\tilde{\sigma}_c^2 = \tilde{\sigma}_e^2 = \tilde{\sigma}^2$, when $n_{\min} = 1$ and $n_{\max} = 1000$.

		$\tau = 0$		$\tau = \delta$	
σ^2	$\tilde{\sigma}^2$	Blinded	Unblinded	Blinded	Unblinded
Trial Design Setting 1					
0.25	0.125	0.0502	0.0507	0.8980	0.8980
0.25	0.25	0.0510	0.0518	0.9000	0.8990
0.25	0.375	0.0511	0.0515	0.8990	0.8990
0.5	0.25	0.0496	0.0516	0.8990	0.8980
0.5	0.5	0.0521	0.0513	0.8990	0.9000
0.5	0.75	0.0512	0.0512	0.9000	0.9020
1	0.5	0.0524	0.0508	0.8970	0.9000
1	1	0.0507	0.0512	0.8990	0.8990
1	1.5	0.0513	0.0512	0.9000	0.9000
2.5	1.25	0.0510	0.0514	0.8990	0.8970
2.5	2.5	0.0511	0.0513	0.8990	0.9000
2.5	3.75	0.0530	0.0523	0.9000	0.9020
5	2.5	0.0512	0.0521	0.8980	0.8980
5	5	0.0499	0.0502	0.8990	0.9010
5	7.5	0.0513	0.0526	0.8990	0.9000
10	5	0.0517	0.0500	0.8970	0.8970
10	10	0.0516	0.0520	0.9010	0.9000
10	15	0.0502	0.0506	0.8980	0.9010
Trial Design Setting 2					
0.25	0.125	0.0249	0.0254	0.8080	0.8030
0.25	0.25	0.0247	0.0255	0.8060	0.8070
0.25	0.375	0.0254	0.0252	0.8100	0.8110
0.5	0.25	0.0253	0.0252	0.8050	0.8050
0.5	0.5	0.0252	0.0261	0.8070	0.8080
0.5	0.75	0.0254	0.0251	0.8110	0.8110
1	0.5	0.0248	0.0255	0.8070	0.8040
1	1	0.0251	0.0260	0.8080	0.8080
1	1.5	0.0259	0.0252	0.8100	0.8100
2.5	1.25	0.0245	0.0255	0.8060	0.8040
2.5	2.5	0.0256	0.0254	0.8070	0.8040
2.5	3.75	0.0253	0.0248	0.8120	0.8100
5	2.5	0.0253	0.0252	0.8060	0.8050
5	5	0.0252	0.0251	0.8060	0.8080
5	7.5	0.0251	0.0257	0.8100	0.8090
10	5	0.0256	0.0238	0.8050	0.8060
10	10	0.0248	0.0255	0.8070	0.8070
10	15	0.0259	0.0252	0.8100	0.8080
